# A Metabolomics-Based Strategy for the Mechanism Exploration of Traditional Chinese Medicine:* Descurainia sophia* Seeds Extract and Fractions as a Case Study

**DOI:** 10.1155/2017/2845173

**Published:** 2017-08-28

**Authors:** Ning Zhou, Ya-Ping Sun, Xiao-Ke Zheng, Qiu-Hong Wang, Yan-Yun Yang, Zhi-Yao Bai, Hai-Xue Kuang, Wei-Sheng Feng

**Affiliations:** ^1^Key Laboratory of Chinese Materia Medica, Heilongjiang University of Chinese Medicine, Harbin 150040, China; ^2^College of Pharmacy, Henan University of Chinese Medicine, Zhengzhou 450046, China; ^3^School of Traditional Chinese Medicine, Guangdong Pharmaceutical University, Guangzhou 510224, China

## Abstract

A UPLC-QTOF-MS based metabolomics research was conducted to explore potential biomarkers which would increase our understanding of the model and to assess the integral efficacy of* Descurainia sophia* seeds extract (DS-A). Additionally, DS-A was split into five fractions in descending order of polarity, which were utilized to illustrate the mechanism together. The 26 identified biomarkers were mainly related to disturbances in phenylalanine, tyrosine, tryptophan, purine, arginine, and proline metabolism. Furthermore, heat map, hierarchical cluster analysis (HCA), and correlation network diagram of biomarkers perturbed by modeling were all conducted. The results of heat map and HCA suggested that fat oil fraction could reverse the abnormal metabolism in the model to some extent; meanwhile the metabolic inhibitory effect produced by the other four fractions helped to relieve cardiac load and compensate the insufficient energy supplement induced by the existing heart and lung injury in model rats. Briefly, the split fractions interfered with the model from different aspects and ultimately constituted the overall effects of extract. In conclusion, the metabolomics method, combined with split fractions of extract, is a powerful approach for illustrating pathologic changes of Chinese medicine syndrome and action mechanisms of traditional Chinese medicine.

## 1. Introduction

Traditional Chinese medicine (TCM) is characterized by its complex composition and complicated mechanism. The absence of appropriate research method leads to the fact that the mechanisms of most TCM are difficult to clarify. Previous studies on the relationship between chemical composition and therapeutic effect are based on either one type of compound, for example, flavonoids [1], alkaloids, and triterpenoids [2], or a total extract [3]. Neither of the above could fully reflect the characteristics of every type of compound in TCM and the contributions to the overall efficacy. In the present study, we employed a novel fraction method based on compound polarity and type to split the extract. As a representative of TCM, the aqueous extract of* Descurainia sophia* seeds (DS-A) was split into five fractions in descending order of polarity: DS-A1 DS-A2, DS-A3, DS-A4, and DS-A5 (the precipitate from water eluted fraction precipitated with ethanol; the supernatant from water eluted fraction precipitated with ethanol; 20% ethanol eluted fraction; 80% ethanol eluted fraction; fat oil fraction extracted by petroleum ether, resp.). Polysaccharides were the main component in DS-A1 fraction, while oligosaccharides were the main component in DS-A2 fraction. Moreover, our group had isolated and identified various monomeric compounds from the other three fractions, including flavonoids, isothiocyanates, thioglycosides, and other chemical composition [4, 5].

*Descurainia sophia* (L.) Webb ex Prantl (flixweed) is a member of family Brassicaceae, which is widely distributed in Asia, Europe, northern Africa, and North America. The seeds of* Descurainia sophia* have been used as a TCM to relieve cough and asthma, promote urination, alleviate edema, and enhance cardiac function for a long time [6]. However, the mechanism and material basis of its efficacy are not yet clear. Previous pharmacological studies have showed that DS-A had excellent performance in “harmful fluid retention in the upper jiao” (R-UJ) model [7], which is a Chinese medicine syndrome model characterized by cough, asthma, chest tightness, and palpitation [8]. Therefore, the R-UJ model was adopted to evaluate the therapeutic effects of DS-A and its fractions.

In the mechanism study, the choices of evaluation method are also critical for the accuracy and reliability of results. It is worth noting that the changes of several biochemical indicators could only reflect partial results in comprehensive actions of TCM, but not the whole. The emergence of metabolomics provides a perfect solution to this problem with its unique merit [9]. Environmental change, drug effect, and other exogenous stimulations always lead to variation in metabolic network of endogenous metabolites, mainly reflected on the metabolite species and quantity. And metabolomics could achieve the overall effect of stimulation on the body through the comprehensive and systematic detection and analysis of endogenous small molecule metabolites in biological samples. Therefore, metabolomics describes the physiological and pathological status from an overall level and offers an effective way to understand the mechanism of TCM [10].

In this manuscript, we utilized R-UJ and DS-A as pathological model and model drug, respectively. Firstly, DS-A was split into five fractions in descending order of polarity: DS-A1, DS-A2, DS-A3, DS-A4, and DS-A5. Then, the metabolomics method was applied to research the influence of DS-A and its five fractions on the metabolic network in the pathological model, thereby proposing a new approach for the mechanism study of TCM.

## 2. Materials and Methods

### 2.1. Chemicals and Reagents

Acetonitrile (HPLC grade) was purchased from Fisher Chemical (USA). Deionized water was prepared with Molecular Water Purification system. Formic acid (LCMS grade, FA) was purchased from Anaqua Chemicals Supply Inc. (USA). Isoprenaline hydrochloride (ISO, batch number BCBC7466V) was purchased from Sigma-Aldrich (USA). DS were purchased from Zhengzhou Chinese herbal medicine market (Henan, China). The mentioned herb was authenticated by Professor Suiqing Chen and Chengming Dong; voucher specimens were deposited at the Henan University of Chinese Medicine.

### 2.2. Extraction and Fraction

The dried DS (15 kg) were decocted with water for three times (150 L × 3, 50 min each time) at 100°C. The combined decoction was concentrated and dried in vacuum to obtain DS-A and then chromatographed on a Diaion HP-20 column (15 × 120 cm) and eluted with H_2_O, 20% (v/v) and 80% ethanol to obtain the water eluted fraction, 20% ethanol eluted fraction (DS-A3), and 80% ethanol eluted fraction (DS-A4), respectively. Subsequently, the water eluted fraction was precipitated with 95% ethanol to obtain the precipitate (DS-A1) and supernatant (DS-A2). On the other side, DS-A was extracted with petroleum ether to obtain the fat oil fraction (DS-A5). The base peak chromatograms of DS-A and its fractions by UPLC-QTOF/MS in ESI^−^ and ESI^+^ mode are shown in Figures S. 1(a) and 1(b), in Supplementary Material available online at https://doi.org/10.1155/2017/2845173.

### 2.3. Animals Handling

Wistar rats (weighing 200 ± 20 g, male and female in half) were obtained from the Laboratory Animal Center of Shandong Lukang Pharmaceutical Co. Ltd. (China). All animals were housed at 20 ± 2°C with a 12 h light/12 h dark cycle and free access to water and food. All animal experiments were performed in accordance with institutional guidelines and ethics.

Eighty rats were randomly divided into 8 groups: control group (C), R-UJ model group (R-UJ), and six DS-treated groups receiving different fraction of DS after modeling. DS-A group received aqueous extract of DS (404.6 mg/kg/d); DS-A1~DS-A5 group received DS-A1~DS-A5 fraction (121.8 mg/kg/d, 238.9 mg/kg/d, 45.7 mg/kg/d, 55.6 mg/kg/d, and 754.6 mg/kg/d), respectively. The drug was orally administered once a day for 4 weeks; C and R-UJ group were orally given with water in the meanwhile. All animals were sacrificed after a collection of 24 h urine samples with metabolic cages. Subsequently, we collected blood from the abdominal aorta then removed and processed the heart and lung to detect the tissue injury.

R-UJ model was induced by subcutaneous (s.c.) injection of ISO at the dose of 20 mg/kg (day 1), 10 mg/kg (day 2), 5 mg/kg (day 3), and 3 mg/kg/d (day 4–20). Two weeks later, tracheal intubation was performed. Then the rats were placed in cold environment for 7 days (4°C, 4 h/d) [8].

### 2.4. Biochemical and Histological Assessment

Heart and lung injury of rats were estimated by heart coefficient (heart weight/body weight, g/100 g) and lung coefficient (lung weight/body weight, g/100 g). Part of the fresh heart and lung tissue were rapidly put into 10% formalin solution for tissue slices preparation. Hematoxylin-eosin (HE) staining sections of heart and lung were observed under microscope (ECLIPSE TS100, Nikon, Japan).

### 2.5. Sample Preparation

Urine samples were stored at −80°C before being analyzed by UPLC-QTOF-MS. Prior to the analysis, urine samples were thawed in ice-water and then centrifuged at 4°C (20,000*g* for 10 min). Each 300 *μ*L aliquot of the supernatant was mixed with 900 *μ*L cold acetonitrile. The mixture was vortexed for 3 min and centrifuged at 20,000*g* for 10 min; then 2 *μ*L of the supernatant was injected into the UPLC.

### 2.6. UPLC-QTOF-MS Analysis of Urine

Separation was performed by UPLC (Dionex UltiMate 3000 System, Thermo Scientific, USA) and screened with ESI-MS. The LC system was comprised of an Acclaim™ RSLC 120 C_18_ column (2.2 *µ*m, 2.1 × 100 mm; Thermo Scientific, USA). The mobile phase was composed of solvent A (0.1% formic acid-water) and solvent B (acetonitrile) with a gradient elution (0-1 min, 98–90% A; 1–9 min, 90–80% A; 9–16 min, 80–70% A; 16–20 min, 70–2% A). The flow rate of mobile phase was 0.3 mL/min. The column temperature was maintained at 40°C, and the sample manager temperature was set at 4°C.

Mass spectrometry was performed on a Quadrupole-Time of Flight Mass Spectrometer (QTOF-MS; maXis HD, Bruker, Germany) using an ESI source. The scanning mass range (*m*/*z*) was from 50 to 1500 with spectra rate of 1.00 Hz. The capillary voltage was set at 3500 V and 3200 V (positive and negative mode, resp.). The pressure of the nebulizer was set at 2.0 Bar, the dry gas temperature at 230°C, and the continuous dry gas flow rate at 8 L/min.

At the beginning of the sequence, we ran five quality control (QC) samples to avoid small changes in both chromatographic retention time and signal intensity. The QC samples were also injected at regular intervals (every six samples) throughout the analytical run.

### 2.7. Statistical Analysis

The raw data were calibrated, peak aligned, background noise subtracted, and normalized by Profile Analysis (version 2.1, Bruker, Germany). The consequent “bucket table” was imported into the SIMCA-P software (version 13.0 Umetrics AB, Sweden) for multivariate analysis. A Principal Component Analysis (PCA) was first applied as an unsupervised method for data visualization and outlier identification [11]. Supervised regression modeling was then performed by Orthogonal Partial Least Squares Discriminant Analysis (OPLS-DA) to identify potential biomarkers. The biomarkers were filtered by the results of variable importance for the projection (VIP) values (VIP > 1.5) and *t*-test (*P* < 0.05). *R*^2^ and *Q*^2^ values are important indicators to assess the quality of fitting model. *R*^2^ displays the variance in the model, indicating the quality of the fitting. *Q*^2^ displays the variance of the data, indicating the model's predictability.

Furthermore, heat map and HCA were conducted by MeV software (version 4.8.0.). The correlation network was constructed based on MetaboAnalyst (http://www.metaboanalyst.ca/), KEGG (http://www.kegg.jp/), and MBRole database (http://csbg.cnb.csic.es/mbrole2) [12].

## 3. Results

### 3.1. Biochemical Analysis and Histopathological Observations

**Figure 1:**
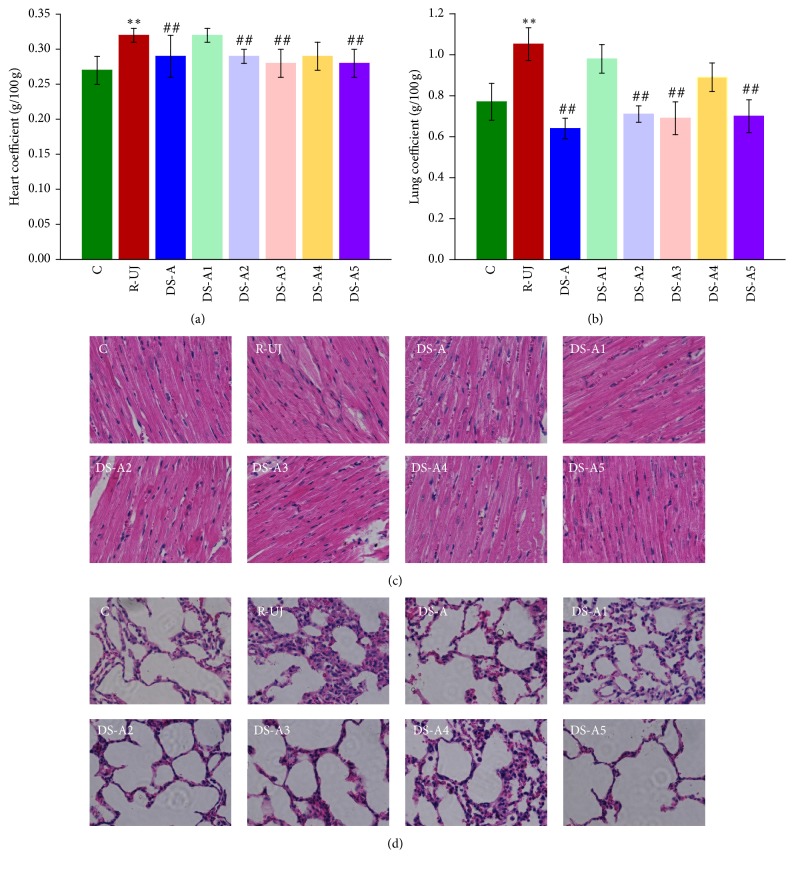
Organ coefficients and histopathological examination (magnification 400x) in C, R-UJ model, and DS-treated groups: heart coefficient (a), lung coefficient (b), HE stained slices of heart (c), and lung (d). ^*∗∗*^*P* < 0.01, compared with the control group; ^##^*P* < 0.01, compared with the model group.

As shown in Figures 1(a) and 1(b), the organ coefficients of model group were significantly (*P* < 0.01) higher than that of the C group, indicating the appearance of heart and lung injury after modeling. In the six DS-treated groups, the injury of heart and lung was significantly (*P* < 0.01) improved in the DS-A, DS-A2, DS-A3, and DS-A5 group.

A similar phenomenon also appeared in the result of histopathological examination, as shown in Figures 1(c) and 1(d). Compared with C group, cardiac hypertrophy and pulmonary interstitial hyperplasia were evident in model group. In the six DS-treated groups, the heart and lung injury was repaired significantly (*P* < 0.01) in the DS-A, DS-A3, and DS-A5 group.

### 3.2. Metabolic Profiling of Urine

**Figure 2:**
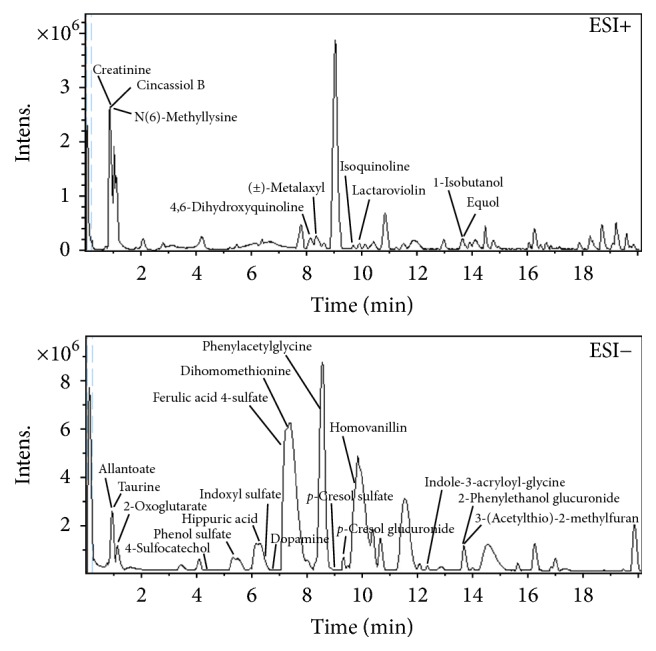
Representative base peak chromatograms obtained from urine in ESI^+^ and ESI^−^ mode.

Chromatographic parameters, such as gradient of mobile phase, flow rate, column temperature, and injection volume, were all optimized for urine sample analysis. The best peak shapes and resolution obtained are shown in Figure 2.

QC samples were run in both negative and positive mode at regular intervals (every six samples) throughout the entire sequence to monitor the stability of the LCMS system. The RSDs of peak areas and retention times of the potential biomarkers in extracted ion chromatogram were calculated. More than 90% of the RSDs were less than 30%. Therefore, the precision and repeatability of the system were highly acceptable.

**Figure 3:**
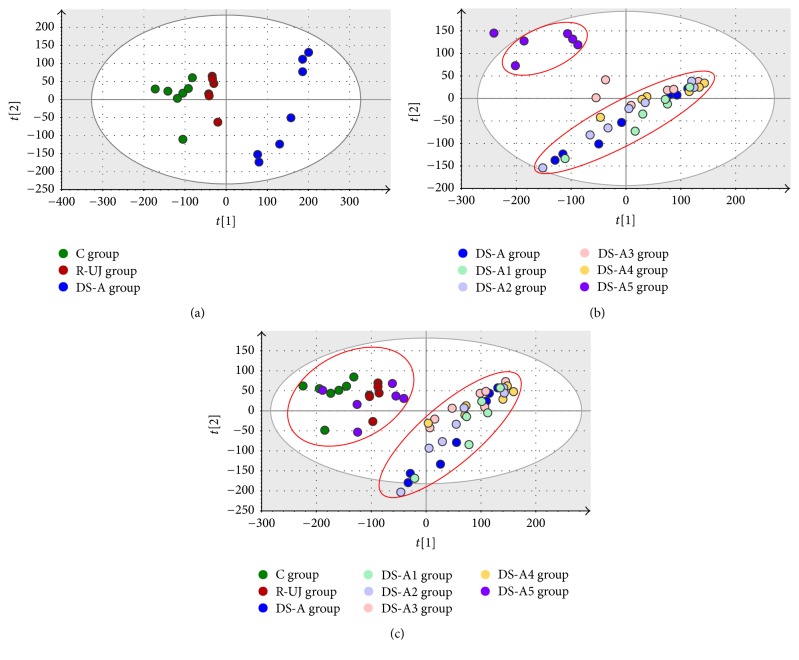
Multivariate data analysis: PCA score scatter plot obtained from C, R-UJ model, and DS-A group (a); PCA score scatter plot obtained from all DS-treated groups (b); PCA score scatter plot obtained from all groups (c).

The normalized data of ESI^−^ and ESI^+^ were merged and imported into SIMCA-P software for multivariate statistical analysis. PCA was first used to investigate the entire metabolic variations in model and DS-treated groups. Firstly, a clear grouping trend is exhibited (*R*^2^*X* = 0.678; *Q*^2^ = 0.444) between C, R-UJ, and DS-A group, as shown in Figure 3(a). The observation indicated that modeling disturbed metabolism of endogenous substances, and they deviated from the normal state. DS-A had effect on R-UJ model rats, although the trajectory did not return to baseline value. In order to reveal the contributions from different fractions of DS-A, we analyzed all the DS-treated groups. The results exhibited an obvious grouping trend (*R*^2^*X* = 0.582; *Q*^2^ = 0.346) between DS-A5 and the other four fraction groups (DS-A1, DS-A2, DS-A3, and DS-A4), as shown in Figure 3(b). It revealed that the effects of DS-A might be the combined effects of its five fractions. Furthermore, a PCA was performed for all groups which confirmed our reasoning. As shown in Figure 3(c), C, R-UJ, and DS-A5 group close together as one category; DS-A and the other four fraction treated groups close together as the other category (*R*^2^*X* = 0.613; *Q*^2^ = 0.441). Also, the result of HCA was consistent with PCA.

### 3.3. Potential Biomarkers

**Figure 4:**
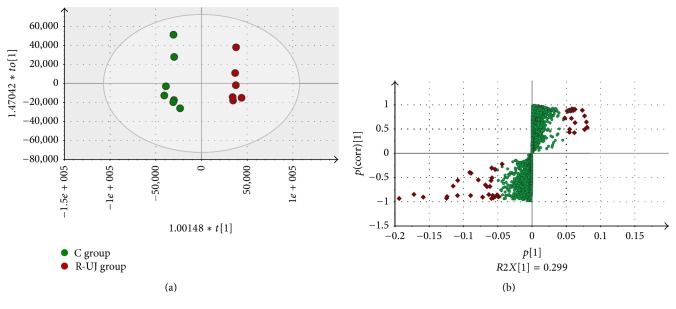
OPLS-DA score scatter plot obtained from C versus R-UJ model group (a); S-plot of OPLS-DA for R-UJ model group (b). Red diamonds in (b) refer to significantly changed metabolites in urine. Green round points in (b) refer to the other metabolites in urine.

The supervised OPLS-DA model was established to compare the metabolic changes between C and R-UJ group. As shown in OPLS-DA score scatter plot, a clear separation was observed based on the first two components (Figure 4(a); *R*^2^*X* = 0.723; *R*^2^*X* = 0.983; *Q*^2^ = 0.824). Before being approved as potential biomarkers, the significantly changed metabolites were carefully screened by VIP values (VIP > 1.5) and *t*-test (*P* < 0.05), as shown in Figure 4(b).

**Figure 5:**
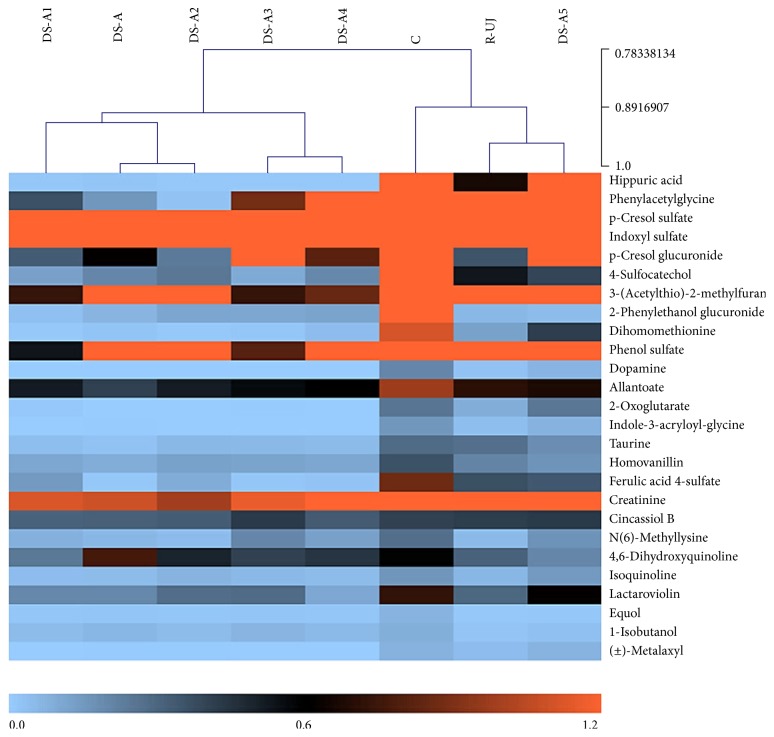
Heat map of the 26 potential biomarkers in C, R-UJ, DS-A, DS-A1, DS-A2, DS-A3, DS-A4, and DS-A5 group. The colors changing from blue to orange indicate more metabolites.

The structures of metabolites were then identified according to the online database such as Metlin (https://metlin.scripps.edu/), Human Metabolome Database (http://www.hmdb.ca/), and MassBank (http://www.massbank.jp/) using the data of accurate mass, MS/MS fragment, and the origin. Further confirmation was acquired by comparing the retention time and MS/MS fragment pattern with authentic standards when it was necessary [13]. Consequently, a total of 26 potential biomarkers of R-UJ rats, including 17 in ESI^−^ and 9 in ESI^+^ mode, were identified and listed in Table 1.  Figure 5 is a heat map showing the average normalized quantities of the 26 metabolites in C, R-UJ, DS-A, DS-A1, DS-A2, DS-A3, DS-A4, and DS-A5 group. Nearly all the biomarkers showed a significantly decreasing change (*P* < 0.05) in R-UJ group compared to C group. Only DS-A5 group exhibited a reverse to normal status; the other four fractions (DS-A1, DS-A2, DS-A3, and DS-A4) seemed to exacerbate this decline. But the metabolic inhibitory effect produced by the four of them might involve other metabolism in the body and play therapeutic effect from a different aspect.

### 3.4. Correlation Network of Differential Metabolites

To investigate the latent relationships between the metabolites, a correlation network diagram was constructed based on MetaboAnalyst, KEGG, and MBRole databases. All the 26 biomarkers were imported into the MBRole database to obtain the categorical annotations (*P* < 0.05). As shown in Table 2, there are mainly three enriched metabolic pathways, including five highlighted metabolites of hippuric acid, phenylacetylglycine, dopamine, homovanillin, and taurine which provided the key information for constructing the network diagram.

**Figure 6:**
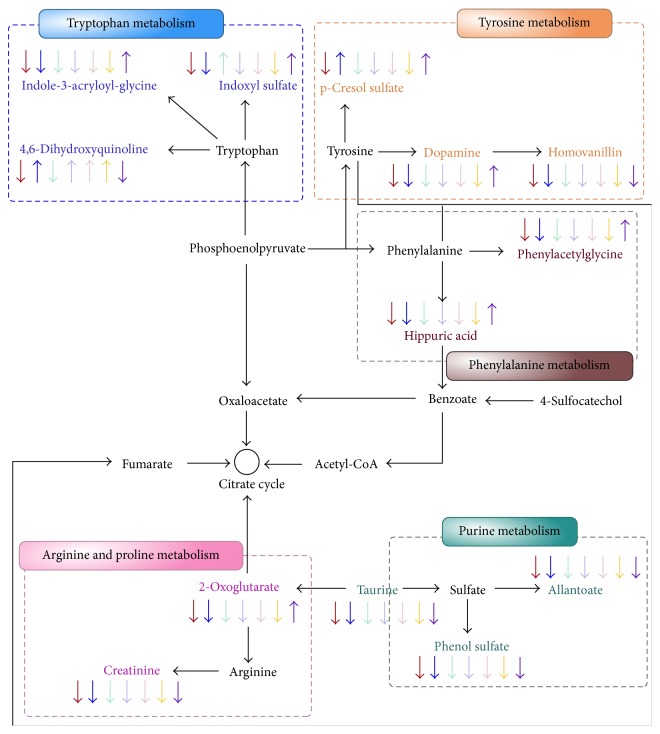
Metabolic network of the significantly changed metabolites in urine of R-UJ rat. 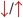 represents R-UJ model group compared with the control group; 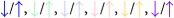 represents DS-A, DS-A1, DS-A2, DS-A3, DS-A4, and DS-A5 group compared with R-UJ model group, respectively.

Consequently, a metabolic pathway map including significantly changed metabolites in urine of R-UJ rats was constructed based on KEGG database and relevant literatures. As shown in Figure 6, five metabolic pathways perturbed by modeling, including phenylalanine metabolism, tyrosine metabolism, tryptophan metabolism, purine metabolism, arginine, and proline metabolism, were related to each other via the citrate cycle.

## 4. Discussion

### 4.1. The R-UJ Model

The R-UJ model is a typical Chinese medicine symptom model, which is suitable for researching the mechanism of DS—a classical TCM used for heart and lung diseases all long time [6]. According to literature method, R-UJ model was induced by s.c. injection of ISO combined with tracheal intubation and cold stimulus [8]. ISO is a beta-receptor agonist; excessive use will increase myocardial contractility and oxygen consumption and finally result in compensatory cardiac hypertrophy [14]. Tracheal intubation increased lung ventilation; if combined with cold stimulus large amount of cold air could cause pulmonary interstitial hyperplasia and alveolar diffuse edema [15]. The results of biochemical analysis and histopathological observations, together with the corresponding symptoms such as cough, asthma, and cardiac insufficiency appearing in model group all confirmed that the R-UJ model was successfully simulated. Since the histopathologic examination was performed four weeks later, the edema may have been absorbed but pulmonary interstitial hyperplasia was still evident.

### 4.2. The Impacts on Metabolism

Results of biochemical indicators and histopathological examination have showed that DS-A could improve the symptoms of R-UJ rats profoundly, while we do not know how DS-A works. Metabolomics study operates a global metabolic profile analysis that matches tightly with the holistic view of Chinese medicine, making it be an effective way for the mechanism research of TCM.

#### 4.2.1. Phenylalanine Metabolism

Phenylalanine (Phe) is known to be a precursor for both hippuric acid (HA) and phenylacetylglycine (PAG), its two major metabolic alterations [16, 17]. The contents of HA and PAG in the urine of model group decreased, indicating that the levels of HA and PAG in plasma declined. So, it is likely that the Phe is not metabolized completely and accumulates in the body. Krause et al. observed an inverse relationship between dopamine excretion and plasma Phe level, which confirmed our reasoning [18], because we detected the decreased excretion of dopamine in model rats. High level of Phe will promote the secretion of insulin on one hand, which lowers the blood glucose level and results in the insufficient energy supply for cardiomyocyte [19], and inhibit the activity of Na^+^, K^+^-ATPase on the other hand [20]. Na^+^, K^+^-ATPase is essential for the maintenance of cardiac function [21] and plays a key role in the regulation of cardiovascular function [22]. It provides energy for myocardial contraction and relaxation and maintains the balance of sodium and potassium ion. In addition, Na^+^, K^+^-ATPase is an important signal transducer in repairing lung injury [23]. Therefore, decreased activity of Na^+^, K^+^-ATPase may aggravate the injury of heart and lung. As shown in Table 1, HA and PAG excretion increased after administration of DS-A5, suggesting that DS-A5 fraction may improve the cardiopulmonary function by promoting Phe metabolism.

#### 4.2.2. Catecholamine Metabolism

Dopamine (DA) is a kind of catecholamine; its effect depends on where it is. DA in plasma could improve urination and renal function [24], which is beneficial to heart. If absorbed by heart, DA would speed up the heart rate, enhance myocardial contractility, increase conduction velocity and cardiac output, and finally result in compensatory cardiac hypertrophy through binding to *β*-2 receptor. As shown in Table 1, the urine content of DA decreased in the model group, which may be attributed to the uptake of DA by heart and the consequent low level of DA in plasma. As a result, the heart suffered double damage. After administration of DS-A5, DA excretion as well as closely related plasma level of DA increased. Therefore, DS-A5 could reduce the cardiac load and eliminate pulmonary edema by upregulating DA plasma level.

#### 4.2.3. Taurine Metabolism

Taurine (Tau), a ubiquitous endogenous sulfur-containing amino acid, possesses numerous pharmacological and physiological actions, such as antioxidant activity and modulation of calcium homeostasis, against catecholamine and angiotensin II [25], improving cardiac energy metabolism [26]. Oxidative stress leads to impaired contractile function, calcium mishandling, cell death, and ventricular remodeling; adrenochrome induces cardiomyocyte apoptosis [27]; angiotensin II enhances the release of aldosterone, which acts on the kidney to promote water and salt retention. This action contributes to an increase in cardiac preload by increasing body fluid and exacerbates the heart failing. However, the actions of Tau could impact the adverse effects of all the above, making it be a kind of “cardioprotectant.” Also, Tau could enhance glucose utilization in heart without affecting oxygen consumption, suggesting that it may promote a shift in metabolic fuel utilization [28]. In conclusion, Tau plays a key role in modulating both cardiac function and energy metabolism. As shown in Table 1, Tau in the urine of model group was lower than the control group, suggesting that the in vivo content of Tau in the model group decreased and was insufficient for the protecting heart. Corresponding symptoms appearing in model rats also confirmed our reasoning. Tau excretions all decreased in DS-treated groups, indicating that Tau was kept in the body to protect heart after administration of DS-A or its fractions. Effect of DS-A on Tau excretion also explained why DS-A enhanced cardiac function without increasing myocardial oxygen consumption [29].

#### 4.2.4. The Impact on Renal and Cardiac Toxicity

*p*-Cresol sulfate (pCS) is the sulfate conjugate of *p*-cresol, which is formed by microbes from tyrosine; indoxyl sulfate (IS) is the sulfate conjugate of indoxyl, which is formed by microbes from tryptophan [30]. *p*-Cresol glucuronide (pCG) is the glucuronic acid conjugate of *p*-cresol in the intestinal wall [31]. pCS and pCG are uremic solutes; pCS and IS have renal and cardiac toxicity. Thereby, the decreased contents of the three of them in the urine of model group may be due to their accumulation in vivo [32], which would lead to a series of problems. Firstly, pCS and IS could induce significant cellular inflammation reaction, which is an important pathological mechanism for kidney injury [33, 34]; secondly, they promote kidney fibrosis and accelerate kidney disease and renal dysfunction [35–37]; thirdly, they promote cardiomyocyte apoptosis via NAPKH oxidase [38] and cardiac hypertrophy via AM *p*-activated protein kinase/uncoupling protein 2, respectively [39, 40]. As shown in Table 1, the excretion of pCS, pCG, and IS increased after administration of DS-A5, indicating that DS-A5 could restore renal function and improve myocardial injury indirectly by accelerating the excretion of renal and cardiac toxin. Moreover, the diuretic effect benefitting from the improvement in renal function could reduce the cardiac load and eliminate pulmonary edema.

## 5. Conclusion

A UPLC-QTOF-MS based urine metabolomics study was successfully performed to explore potential biomarkers in R-UJ model and investigate the mechanism of DS-A. With the help of biochemical and histological assessment, the model of R-UJ and the efficiency of DS-A were confirmed. The results of PCA, HCA, and heat map suggested that the improvement of cardiac function and elimination of edema in model should be attributed to fat oil fraction (DS-A5), which promoted Phe metabolism, increased plasma level of DA, decreased excretion of Tau, and accelerated excretion of renal and cardiac toxin. Meanwhile, the metabolic inhibitory effect produced by the other four fractions (DS-A1, DS-A2, DS-A3, and DS-A4) helped to relieve cardiac load and compensate the insufficient energy supplement induced by the existing heart and lung injury in model rats. Briefly, the split fractions interfered with the model from different aspects and ultimately constituted the overall effects of extract. In conclusion, the metabolomics method combined with split fractions of extract is a powerful approach for illustrating the pathologic changes of Chinese medicine syndrome and action mechanisms of TCM.

## Highlights

1. A novel fraction method based on compound polarity and type was employed to split the extract of traditional Chinese medicine.
2. The metabolomics approach and split fractions of extract were utilized in combination to illustrate pathologic changes of Chinese medicine syndrome and action mechanisms of traditional Chinese medicine.

## Supplementary Material

Base peak chromatograms of DS-A and its five fractions (DS-A1, DS-A2, DS-A3, DS-A4, DS-A5) in ESI- mode (a) and ESI+ mode (b).

## Figures and Tables

**Table 1:** Potential biomarkers related to R-UJ model.

Mode	Number	Name	Formula	Determined *m/z*	Ion form	*t*_*R*_ (min)	Trend
ESI^−^	1	Hippuric acid	C_9_H_9_NO_3_	178.0510	[M − H]^−^	6.4	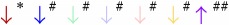
	2	Phenylacetylglycine	C_10_H_11_NO_3_	192.0665	[M − H]^−^	8.4	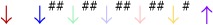
	3	*p*-Cresol sulfate	C_7_H_8_O_4_S	187.0070	[M − H]^−^	9.0	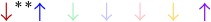
	4	Indoxyl sulfate	C_8_H_7_NO_4_S	212.0021	[M − H]^−^	6.6	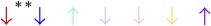
	5	*p*-Cresol glucuronide	C_13_H_16_O_7_	283.0825	[M − H]^−^	9.4	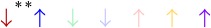
	6	4-Sulfocatechol	C_6_H_6_O_5_S	188.9858	[M − H]^−^	4.4	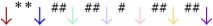
	7	3-(Acetylthio)-2-methylfuran	C_7_H_8_O_2_S	201.0224	[M + FA − H]^−^	13.7	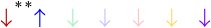
	8	2-Phenylethanol glucuronide	C_14_H_18_O_7_	297.0979	[M − H]^−^	13.7	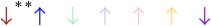
	9	Dihomomethionine	C_7_H_15_NO_2_S	222.0802	[M + FA − H]^−^	7.4	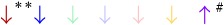
	10	Phenol sulfate	C_6_H_6_O_4_S	172.9911	[M − H]^−^	5.4	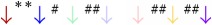
	11	Dopamine	C_8_H_11_NO_2_	134.0603	[M − H_2_O − H]^−^	6.8	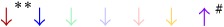
	12	Allantoate	C_4_H_8_N_4_O_4_	157.0361	[M − H_2_O − H]^−^	1.0	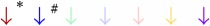
	13	2-Oxoglutarate	C_5_H_6_O_5_	145.0134	[M − H]^−^	1.2	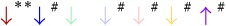
	14	Indole-3-acryloyl-glycine	C_13_H_14_N_2_O_4_	243.0770	[M − H_2_O − H]^−^	12.4	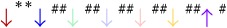
	15	Taurine	C_2_H_7_NO_3_S	124.0065	[M − H]^−^	1.0	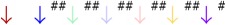
	16	Homovanillin	C_9_H_10_O_3_	165.0551	[M − H]^−^	9.8	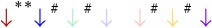
	17	Ferulic acid 4-sulfate	C_10_H_10_O_7_S	273.0069	[M − H]^−^	7.2	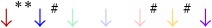

ESI^+^	18	Creatinine	C_4_H_7_N_3_O	114.0664	[M + H]^+^	1.0	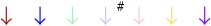
	19	Cincassiol B	C_20_H_32_O_8_	212.1033	[M + Na + H]^2+^	1.0	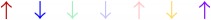
	20	N(6)-Methyllysine	C_7_H_16_N_2_O_2_	143.1181	[M − H_2_O + H]^+^	1.0	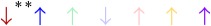
	21	4,6-Dihydroxyquinoline	C_9_H_7_NO_2_	162.0550	[M + H]^+^	8.2	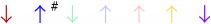
	22	Isoquinoline	C_9_H_7_N	130.0651	[M + H]^+^	9.7	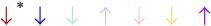
	23	Lactaroviolin	C_15_H_14_O	233.0923	[M + Na]^+^	9.8	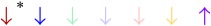
	24	Equol	C_15_H_14_O_3_	243.1018	[M + H]^+^	13.6	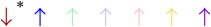
	25	1-Isobutanol	C_21_H_27_NO_10_	436.1603	[M − H_2_O + H]^+^	13.6	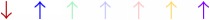
	26	(±)-Metalaxyl	C_15_H_21_NO_4_	280.1543	[M + H]^+^	8.4	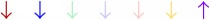

^*∗*^ *P* < 0.05 and ^*∗∗*^*P* < 0.01, compared with the control group; ^#^*P* < 0.05 and ^##^*P* < 0.01, compared with the model group. 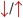 represents R-UJ model group compared with the control group; 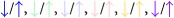 represents DS-A, DS-A1, DS-A2, DS-A3, DS-A4, and DS-A5 group compared with R-UJ model group, respectively.

**Table 2:** Pathway enrichment analysis of perturbed metabolites in R-UJ rats based on MBRole database.

Label	*P *value^a^	Related compounds
Phenylalanine metabolism	0.006	Phenylacetylglycine, hippuric acid
Tyrosine metabolism	0.015	Dopamine, homovanillin
Neuroactive ligand-receptor interaction	0.041	Dopamine, taurine

^a^ *P* value is obtained from analysis of MBRole.
