# Supplementary material for: A Metabolomics-Based Strategy for the Mechanism Exploration of Traditional Chinese Medicine: Descurainia sophia Seeds Extract and Fractions as a Case Study
Source: Evid Based Complement Alternat Med. 2017 Aug 28;2017:2845173. doi: 10.1155/2017/2845173 (PMC5592412; doi:10.1155/2017/2845173)
Supplement: Supplementary file 1 — Base peak chromatograms of DS-A and its five fractions (DS-A1, DS-A2, DS-A3, DS-A4, DS-A5) in ESI- mode (a) and ESI+ mode (b). [file 2845173.f1.pdf]

A metabolomics-based strategy for the mechanism exploration of traditional Chinese medicine: *Descurainia sophia* seeds extract and fractions as a case study

Ning Zhou [1, 2], Ya-Ping Sun [2], Xiao-Ke Zheng [2], Qiu-Hong Wang [3], Yan-Yun Yang [2], Zhi-Yao Bai [2], Hai-Xue Kuang [1], Wei-Sheng Feng [2]

[1] *Key Laboratory of Chinese Materia Medica, Heilongjiang University of Chinese Medicine, Harbin 150040, China*

[2] *College of Pharmacy, Henan University of Chinese Medicine, Zhengzhou 450046, China*

[3] *School of Traditional Chinese Medicine, Guangdong Pharmaceutical University, Guangzhou 510224, China*

Correspondence should be addressed to Wei-Sheng Feng; fwsh@hactcm.edu.cn, Hai-Xue Kuang; hxkuang56@163.com

Abstract

A UPLC-QTOF-MS based metabolomics research was conducted to explore potential biomarkers which would increase our understanding of the model, and to assess the integral efficacy of *Descurainia sophia* seeds extract (DS-A). Additionally, DS-A was split into five fractions in descending order of polarity, which was utilized to illustrate the mechanism together. The 26 identified biomarkers were mainly related to disturbances in phenylalanine, tyrosine, tryptophan, purine, arginine and proline metabolism. Furthermore, heat map, hierarchical cluster analysis (HCA) and correlation network diagram of biomarkers perturbed by modeling were all conducted. It suggested that fat oil fraction could reverse the abnormal metabolism in the model to some extent, meanwhile the metabolic inhibitory effect produced by the other four fractions helped to relieve cardiac load and compensate the insufficient energy supplement induced by the existing heart and lung injury in model rats. Briefly, the split fractions interfered with the model from different aspects, and ultimately constituted the overall effects of extract. In conclusion, the metabolomics method, combined with split fractions of extract, is a powerful approach for illustrating pathologic changes of Chinese medicine syndrome and action mechanisms of traditional Chinese medicine.

Keywords: Metabolomics; UPLC-QTOF-MS; *Descurainia sophia* seeds; fractions of extract;

1. Introduction

Traditional Chinese medicine (TCM) is characterized by its complex composition and complicated mechanism. The absence of appropriate research method leads to the fact that the mechanisms of most TCM are difficult to clarify. Previous studies on the relationship between chemical composition and therapeutic effect are based on either one type of compound, e.g., flavonoids [1], alkaloids, triterpenoids [2], or a total extract [3]. Neither of above could fully reflect the characteristics of every type of compound in TCM and the contributions to the overall efficacy. In the present study, we employed a novel fraction method based on compound polarity and type to split the extract. As a representative of TCM, the aqueous extract of *Descurainia sophia* seeds (DS-A) was split into five fractions in descending order of polarity: DS-A1 DS-A2, DS-A3, DS-A4, DS-A5 (the precipitate from water eluted fraction precipitated with ethanol; the supernatant from water eluted fraction precipitated with ethanol; 20% ethanol eluted fraction; 80 % ethanol eluted fraction; fat oil fraction extracted by petroleum ether, respectively). Polysaccharides were the main component in DS-A1 fraction, while oligosaccharides in DS-A2 fraction. Moreover, our group have isolated and identified various monomeric compounds from the other three fractions, including flavonoids, isothiocyanates, thioglycosides and other chemical composition [4, 5].

Descurainia sophia (L.) Webb ex Prantl (Flixweed) is a member of family Brassicaceae, which is widely distributed in Asia, Europe, northern Africa and North America. The seeds of *Descurainia sophia* have been used as a TCM to relieve cough and asthma, promote urination, alleviate edema and enhance cardiac function for a long time [6]. However, the mechanism and material basis of its efficacy are not yet clear. Previous pharmacological studies have showed that, DS-A had excellent performance in “harmful fluid retention in the upper jiao” (R-UJ) model [7], which is a Chinese medicine syndrome model characterized by cough, asthma, chest tightness and palpitation [8]. Therefore, the R-UJ model was adopted to evaluate the therapeutic effects of DS-A and its fractions.

In the mechanism study, the choices of evaluation method are also critical for the accuracy and reliability of results. It’s worth noting that the changes of several biochemical indicators could only reflect partial results in comprehensive actions of TCM, while, not the whole. The emergence of metabolomics provides a perfect solution to this problem with its unique merit [9]. Environmental change, drug effect and other exogenous stimulations always lead to variation in metabolic network

of endogenous metabolites, mainly reflected on the metabolite species and quantity. And metabolomics could achieve the overall effect of stimulation on the body through the comprehensive and systematic detection and analysis of endogenous small molecule metabolites in biological samples. Therefore, metabolomics describes the physiological and pathological status from an overall level, and offers an effective way to understand the mechanism of TCM [10].

In this manuscript, we utilized R-UJ and DS-A as pathological model and model drug, respectively. Firstly, DS-A was split into five fractions in descending order of polarity: DS-A1, DS-A2, DS-A3, DS-A4, DS-A5. Then, the metabolomics method was applied to research the influence of DS-A and its five fractions on the metabolic network in the pathological model, thereby proposing a new approach for the mechanism study of TCM.

2. Materials and Methods

2.1. Chemicals and Reagents. Acetonitrile (HPLC grade) was purchased from Fisher Chemical (USA). Deionized water was prepared with Molecular Water Purification system. Formic acid (LCMS grade, FA) was purchased from Anaqua Chemicals Supply Inc (USA). Isoprenaline Hydrochloride (ISO, batch number BCBC7466V) was purchased from Sigma-Aldrich (USA). DS were purchased from Zhengzhou Chinese herbal medicine market (Henan, China). The mentioned herb was authenticated by Professor Suiqing Chen and Chengming Dong, voucher specimens were deposited at the Henan University of Chinese Medicine.

2.2. Extraction and Fraction. The dried DS (15 kg) were decocted with water for three times (150 L × 3, 50 min each time) at 100 °C. The combined decoction was concentrated and dried in vacuum to obtain DS-A, then chromatographed on a Diaion HP-20 column (15 × 120 cm) and eluted with H_2O, 20% (v / v) and 80% ethanol to obtain the water eluted fraction, 20% ethanol eluted fraction (DS-A3) and 80% ethanol eluted fraction (DS-A4) respectively. Subsequently, the water eluted fraction was precipitated with 95% ethanol to obtain the precipitate (DS-A1) and supernatant (DS-A2). On the other side, DS-A was extracted with petroleum ether to obtain the fat oil fraction (DS-A5). The base peak chromatograms of DS-A and its fractions by UPLC-QTOF/MS in ESI^- and ESI^+ mode are shown in Figures S. 1(a) and 1(b).

2.3. Animals Handling. Wistar rats (weighing 200 ± 20 g, male and female in half) were obtained from the Laboratory Animal Center of Shandong Lukang Pharmaceutical Co. Ltd. (China). All animals were housed at 20 ± 2 ^{o}C with a 12 h light / 12 h dark cycle and free access to water and food. All animal experiments were performed in accordance with institutional guidelines and ethics.

Eighty rats were randomly divided into 8 groups: control group (C), R-UJ model group (R-UJ) and six DS-treated groups receiving different fraction of DS after modeling. DS-A group received aqueous extract of DS (404.6 mg/kg/d); DS-A1~DS-A5 group received DS-A1~DS-A5 fraction (121.8 mg/kg/d, 238.9 mg/kg/d, 45.7 mg/kg/d, 55.6 mg/kg/d, 754.6 mg/kg/d) respectively. The drug was orally administered once a day for 4 weeks, C and R-UJ group were orally administered with water in the meanwhile. All animals were sacrificed after a collection of 24 h urine samples with metabolic cages. Subsequently, we collected blood from the abdominal aorta then removed and processed the heart and lung to detect the tissue injury.

R-UJ model was induced by subcutaneous (s.c.) injection of ISO at the dose of 20 mg/kg (day 1), 10 mg/kg (day 2), 5 mg/kg (day 3), 3 mg/kg/d (day 4-20). Two weeks later, tracheal intubation was performed. Then the rats were placed in cold environment for 7 days (4 ^{o}C, 4 h/d) [8].

2.4. Biochemical and Histological Assessment. Heart and lung injury of rats were estimated by heart coefficient (heart weight/body weight, g/100g) and lung coefficient (lung weight/body weight, g/100g). Part of the fresh heart and lung tissue were rapidly put into 10% formalin solution for tissue slices preparation. Hematoxylin-eosin (HE) staining sections of heart and lung were observed under microscope (ECLIPSE TS100, Nikon, Japan).

2.5. Sample Preparation. Urine samples were stored at -80 ^{o}C before being analyzed by UPLC-QTOF-MS. Prior to the analysis, urine samples were thawed in ice-water, then centrifuged at 4 °C (20, 000 g for 10 min). Each 300 μL aliquot of the supernatant was mixed with 900 μL cold acetonitrile. The mixture was vortexed for 3 min and centrifuged at 20,000 g for 10 min, then 2 μL of the supernatant was injected into the UPLC.

2.6. UPLC-QTOF-MS Analysis of Urine. Separation was performed by UPLC (Dionex UltiMate 3000 system, Thermo Scientific, USA) and screened with ESI-MS. The LC system was comprised of

an Acclaim™ RSLC 120 C_{18} column (2.2 μm, 2.1×100 mm; Thermo Scientific, USA). The mobile phase was composed of solvent A (0.1% formic acid-water) and solvent B (acetonitrile) with a gradient elution (0-1 min, 98-90% A; 1-9 min, 90-80% A; 9-16 min, 80-70% A; 16-20 min, 70-2% A). The flow rate of mobile phase was 0.3 mL/min. The column temperature was maintained at 40 °C, and the sample manager temperature was set at 4 °C.

Mass spectrometry was performed on a Quadrupole-Time of Flight Mass Spectrometer (QTOF-MS; maXis HD, Bruker, Germany) using an ESI source. The scanning mass range (m/z) was from 50 to 1500 with spectra rate of 1.00 Hz. The capillary voltage was set at 3500 V and 3200 V (positive and negative mode, respectively). The pressure of the nebulizer was set at 2.0 Bar, the dry gas temperature at 230 °C, and the continuous dry gas flow rate at 8 L/min.

At the beginning of the sequence, we ran five quality control (QC) samples to avoid small changes in both chromatographic retention time and signal intensity. The QC samples were also injected at regular intervals (every six samples) throughout the analytical run.

2.7. Statistical Analysis. The raw data were calibrated, peak aligned, background noise subtracted and normalized by Profile Analysis (version 2.1, Bruker, Germany). The consequent “bucket table” was imported into the SIMCA-P software (version 13.0 Umetrics AB, Sweden) for multivariate analysis. A Principal Component Analysis (PCA) was first applied as an unsupervised method for data visualization and outlier identification [11]. Supervised regression modeling was then performed by Orthogonal Partial Least Squares Discriminant Analysis (OPLS-DA) to identify potential biomarkers. The biomarkers were filtered by the results of variable importance for the projection (VIP) values (VIP > 1.5) and t-test ($P < 0.05$). R^2 and Q^2 values are important indicators to assess the quality of fitting model. R^2 displays the variance in the model, indicating the quality of the fitting. Q^2 displays the variance of the data, indicating the model's predictability.

Furthermore, heat map and HCA were conducted by MeV software (version 4.8.0.). The correlation network was constructed based on Metabo Analyst (http://www.metaboanalyst.ca/), KEGG (http://www.kegg.jp/) and MBRole database (http://csbg.cnb.csic.es/mbrole2) [12].

3. Results

3.1 Biochemical Analysis and Histopathological Observations. As shown in Figures 1(a) and 1(b),

the organ coefficients of model group were significantly (P < 0.01) higher than that of the C group, indicating the appearance of heart and lung injury after modeling. In the six DS-treated groups, the injury of heart and lung was significantly (P < 0.01) improved in the DS-A, DS-A2, DS-A3 and DS-A5 group.

A similar phenomenon also appeared in the result of histopathological examination, as shown in Figures 1(c) and 1(d). Compared with C group, cardiac hypertrophy and pulmonary interstitial hyperplasia were evident in model group. In the six DS-treated groups, the heart and lung injury was repaired significantly (P < 0.01) in the DS-A, DS-A3 and DS-A5 group.

3.2. Metabolic Profiling of Urine. Chromatographic parameters, such as gradient of mobile phase, flow rate, column temperature and injection volume, were all optimized for urine sample analysis. The best peak shapes and resolution obtained are shown in Figure 2.

QC samples were run in both negative and positive mode at regular intervals (every six samples) throughout the entire sequence to monitor the stability of the LC-MS system. The RSDs of peak areas and retention times of the potential biomarkers in extracted ion chromatogram were calculated. More than 90% of the RSDs were less than 30%. Therefore, the precision and repeatability of the system were highly acceptable.

The normalized data of ESI^- and ESI^+ were merged and imported into SIMCA-P software for multivariate statistical analysis. PCA was first used to investigate the entire metabolic variations in model and DS-treated groups. Firstly, there exhibits a clear grouping trend (R^2X = 0.678; Q^2 = 0.444) between C, R-UJ and DS-A group, as shown in Figure 3(a). The observation indicated that modeling disturbed metabolism of endogenous substances, and they deviated from the normal state. DS-A had effect on R-UJ model rats, although the trajectory did not return to baseline value. In order to reveal the contributions from different fractions of DS-A, we analyzed all the DS-treated groups. The results exhibited an obvious grouping trend (R^2X = 0.582; Q^2 = 0.346) between DS-A5 and the other four fraction groups (DS-A1, DS-A2, DS-A3, DS-A4), as shown in Figure 3(b). It revealed that the effects of DS-A might be the combined effects of its five fractions. Furthermore, a PCA was performed for all groups which confirmed our reasoning. As shown in Figure 3(c), C, R-UJ and DS-A5 group close together as one category, DS-A and the other four fraction treated groups close together as the other category (R^2X = 0.613; Q^2 = 0.441). Also, the result of HCA was consistent with

PCA.

3.3. Potential Biomarkers. The supervised OPLS-DA model was established to compare the metabolic changes between C and R-UJ group. As shown in OPLS-DA score scatter plot, a clear separation was observed based on the first two components (Figure 4(a), R^2X = 0.723; R^2X = 0.983; Q^2 = 0.824). Before being approved as potential biomarkers, the significantly changed metabolites were carefully screened by VIP values (VIP > 1.5) and t-test (P < 0.05), as shown in Figure 4(b).

The structures of metabolites were then identified according to the online database such as Metlin (https://metlin.scripps.edu/), Human Metabolome Database (http://www.hmdb.ca/), and MassBank (http://www.massbank.jp/) using the data of accurate mass, MS/MS fragment and the origin. Further confirmation was acquired by comparing the retention time and MS/MS fragment pattern with authentic standards when it was necessary [13]. Consequently, a total of 26 potential biomarkers of R-UJ rats, including 17 in ESI^- and 9 in ESI^+ mode, were identified and listed in Table 1. Figure 5 is a heat map showing the average normalized quantities of the 26 metabolites in C, R-UJ, DS-A, DS-A1, DS-A2, DS-A3, DS-A4 and DS-A5 group. Nearly all the biomarkers showed a significantly decreasing change (P < 0.05) in R-UJ group compared to C group. Only DS-A5 group exhibited a reverse to normal status, the other four fractions (DS-A1, DS-A2, DS-A3, DS-A4) seemed to exacerbate this decline. But it did not rule out that the metabolic inhibitory effect produced by them four might involve other metabolism in the body and play therapeutic effect from a different aspect.

3.4. Correlation network of differential metabolites. To investigate the latent relationships between the metabolites, a correlation network diagram was constructed based on Metabo Analyst, KEGG and MBRole databases. All the 26 biomarkers were imported into the MBRole database to obtain the categorical annotations (P < 0.05). As shown in Table 2, there are mainly three enriched metabolic pathways, including five highlighted metabolites of Hippuric acid, Phenylacetylglycine, Dopamine, Homovanillin, and Taurine which provided the key information for constructing the network diagram.

Consequently, a metabolic pathway map including significantly changed metabolites in urine of R-UJ rats was constructed based on KEGG database and relevant literatures. As shown in Figure 6, five metabolic pathways perturbed by modeling, including phenylalanine metabolism, tyrosine

metabolism, tryptophan metabolism, purine metabolism, arginine and proline metabolism were related to each other via the citrate cycle.

4. Discussion

4.1. The R-UJ model. The R-UJ model is a typical Chinese medicine symptom model, which is suitable for researching the mechanism of DS --- a classical TCM used for heart and lung diseases all long time [6]. According to literature method, R-UJ model was induced by s.c. injection of ISO combined with tracheal intubation and cold stimulus [8]. ISO is a beta receptor agonist, excessive use will increase myocardial contractility and oxygen consumption, and finally result in compensatory cardiac hypertrophy [14]. Tracheal intubation increased lung ventilation, if combined with cold stimulus large number of cold air could cause pulmonary interstitial hyperplasia and alveolar diffuse edema [15]. The results of biochemical analysis and histopathological observations, together with the corresponding symptoms such as cough, asthma and cardiac insufficiency appeared in model group all confirmed that the R-UJ model was successfully simulated. Since the histopathologic examination was performed four weeks later, the edema may have been absorbed but pulmonary interstitial hyperplasia was still evident.

4.2. The impacts on metabolism. Results of biochemical indicators and histopathological examination have showed that DS-A could improve the symptoms of R-UJ rats profoundly. While, we don't know how DS-A works. Metabolomics study operates a global metabolic profile analysis that matches tightly with the holistic view of Chinese medicine, making it to be an effective way for the mechanism research of TCM.

4.2.1. Phenylalanine metabolism. Phenylalanine (Phe) is known to be a precursor for both hippuric acid (HA) and phenylacetylglycine (PAG), its two major metabolic alterations [16, 17]. The contents of HA and PAG in the urine of model group decreased, indicating that the levels of HA and PAG in plasma declined. So, it is likely that the Phe is not metabolized completely and accumulates in the body. Krause et al. observed an inverse relationship between dopamine excretion and plasma Phe level, which confirmed our reasoning [18]. Cause we detected the decreased excretion of dopamine in model rats. High level of Phe will promote the secretion of insulin on one hand, which lowers the

blood glucose level and results in the insufficient energy supply for cardiomyocyte [19], and inhibit the activity of Na^+, K^+-ATPase on the other [20]. Na^+, K^+-ATPase is essential for the maintenance of cardiac function [21], and plays a key role in the regulation of cardiovascular function [22]. It provides energy for myocardial contraction and relaxation, maintains the balance of sodium and potassium ion. In addition, Na^+, K^+-ATPase is an important signal transducer in repairing lung injury [23]. Therefore, decreased activity of Na^+, K^+-ATPase may aggravate the injury of heart and lung. As shown in Table 1, HA and PAG excretion increased after administration of DS-A5, suggesting that DS-A5 fraction may improve the cardio-pulmonary function by promoting Phe metabolism.

4.2.2. Catecholamine metabolism. Dopamine (DA) is a kind of catecholamine, its effect depends on where it is. DA in plasma could improve urination and renal function [24], which is beneficial to heart. If absorbed by heart, DA would speed up the heart rate, enhance myocardial contractility, increase conduction velocity and cardiac output, and finally result in compensatory cardiac hypertrophy through binding to β-2 receptor. As shown in Table 1, the urine content of DA decreased in the model group, which may be attributed to the uptake of DA by heart and the consequent low level of DA in plasma. As a result, the heart suffered double damage. After administration of DS-A5, DA excretion as well as closely related plasma level of DA increased. Therefore, DS-A5 could reduce the cardiac load and eliminate pulmonary edema by up-regulating DA plasma level.

4.2.3. Taurine metabolism. Taurine (Tau), a ubiquitous endogenous sulfur-containing amino acid, possesses numerous pharmacological and physiological actions, such as antioxidant activity, modulation of calcium homeostasis, against catecholamine and angiotensin II [25], improve cardiac energy metabolism [26]. Oxidative stress leads to impaired contractile function, calcium mishandling, cell death and ventricular remodeling; Adrenochrome induces cardiomyocyte apoptosis [27]; Angiotensin II enhances the release of aldosterone, which acts on the kidney to promote water and salt retention. This action contributes to an increase in cardiac preload by increasing body fluid, and exacerbates the heart failing. However, the actions of Tau could impact the adverse effects of all above, making it to be a kind of “cardio-protectant”. Also, Tau could enhance glucose utilization in heart without affecting oxygen consumption, suggesting that it may promote a shift in metabolic fuel utilization [28]. In conclusion, Tau plays a key role in modulating both cardiac function and energy

metabolism. As shown in Table 1, Tau in the urine of model group was lower than the control group, suggesting that the in vivo content of Tau in the model group decreased and was insufficient for the protecting heart. Corresponding symptoms appeared in model rats also confirmed our reasoning. Tau excretions all decreased in DS-treated groups, indicating that Tau was kept in the body to protect heart after administration of DS-A or its fractions. Effect of DS-A on Tau excretion also explained why DS-A enhanced cardiac function without increasing myocardial oxygen consumption [29].

4.2.4. The impact on renal and cardiac toxicity. *p*-Cresol sulfate (pCS) is the sulfate conjugate of *p*-cresol, which is formed by microbes from tyrosine; Indoxyl sulfate (IS) is the sulfate conjugate of indoxyl, which is formed by microbes from tryptophan [30]. *p*-Cresol glucuronide (pCG) is the glucuronic acid conjugate of *p*-cresol in the intestinal wall [31]. pCS and pCG are uremic solutes, pCS and IS have renal and cardiac toxicity. Thereby, the decreased contents of them three in the urine of model group may be due to their accumulation in vivo [32], which would lead to a series of problems. Firstly, pCS and IS could induce significant cellular inflammation reaction, which is an important pathological mechanism for kidney injury [33, 34]; Secondly, they promote kidney fibrosis, accelerate kidney disease and renal dysfunction [35-37]; Thirdly, they promote cardiomyocyte apoptosis via NAPKH oxidase [38], and cardiac hypertrophy via AMP-activated protein kinase / uncoupling protein 2 respectively [39, 40]. As shown in Table 1, the excretion of pCS, pCG and IS increased after administration of DS-A5, indicating that DS-A5 could restore renal function and improve myocardial injury indirectly by accelerating the excretion of renal and cardiac toxin. Moreover, the diuretic effect benefited from the improvement in renal function could reduce the cardiac load and eliminate pulmonary edema.

5. Conclusion

A UPLC-QTOF-MS based urine metabolomics study was successfully performed to explore potential biomarkers in R-UJ model and investigate the mechanism of DS-A. With the help of biochemical and histological assessment, the model of R-UJ and the efficiency of DS-A were confirmed. The results of PCA, HCA and heat map suggested that the improvement of cardiac function and elimination of edema in model should be attributed to fat oil fraction (DS-A5), which promoted Phe metabolism, increased plasma level of DA, decreased excretion of Tau, and

accelerated excretion of renal and cardiac toxin; Meanwhile, the metabolic inhibitory effect produced by the other four fractions (DS-A1, DS-A2, DS-A3, DS-A4) helped to relieve cardiac load and compensate the insufficient energy supplement induced by the existing heart and lung injury in model rats. Briefly, the split fractions interfered with the model from different aspects, and ultimately constituted the overall effects of extract. In conclusion, the metabolomics method combined with split fractions of extract, is a powerful approach for illustrating the pathologic changes of Chinese medicine syndrome and action mechanisms of TCM.

Competing Interests

The authors declare that they have no competing interests.

Authors' Contributions

Ning Zhou and Ya-Ping Sun contributed equally to this work.

Acknowledgments

This study was financially supported by National Basic Research Program of China (973 Program, No. 2013CB531802)

References

[1] Z. Mao, C. Gan, J. Zhu et al., "Anti-atherosclerotic activities of flavonoids from the flowers of *Helichrysum arenarium* L. MOENCH through the pathway of anti-inflammation", *Bioorganic & Medicinal Chemistry Letters*, vol. 2017, Article ID 30453-5, 6 pages, 2017.

[2] H. Xu, Z. Z. Yuan, X. Ma et al., "Triterpenoids with antioxidant activities from Myricaria squamosal", *Journal of Asian Natural Products Research*, vol. 2017, Article ID 1321636, 7 pages, 2017.

[3] J. Y. Choi, C. J. Hwang, H. P. Lee et al., "Inhibitory effect of ethanol extract of Nannochloropsis oceanica on lipopolysaccharide-induced neuroinflammation, oxidative stress, amyloidogensis and memory impairment", *Oncotarget*, vol. 2017, Article ID 17268, 14 pages, 2017.

[4] W. S. Feng, C. G. Li, X. K. Zheng et al., "Three new sulphur glycosides from the seeds of *Descurainia Sophia*", *Natural Product Research*, vol. 30, no. 15, pp. 1675-1681, 2016.

[5] J. H. Gong, Y. L. Zhang, J. L. He et al., "Extractions of oil from *Descurainia sophia* seed using supercritical CO_2, chemical compositions by GC-MS and evaluation of the anti-tussive, expectorant and anti-asthmatic activities", *Molecules*, vol. 20, no. 7, pp. 13296-13312, 2015.

[6] K. Sun, X. Li, J. M. Liu et al., "A novel sulphur glycoside from the seeds of *Descurainia sophia* (L.)", *Journal of Asian Natural Products Research*, vol. 7, no. 6, pp. 853-856, 2005.

[7] Y. P. Sun, S. L. Yang, Y. P. Si et al., "Effect of Aqueous Extract of the seeds of *Descurainia sophia* (L.) Webb ex Prantl. on the Rat Model of Harmful Fluid Retention in the Upper Jiao", *Proceedings of the Thirteenth Youth Symposium on Pharmaceutical Research Achievements, Chinese Pharmaceutical Association*, vol. 4, pp. 489-492, 2016.

[8] W. Xie, X. M. Ji, Z. X. Pang, and S.J. Wang, "Establishment and Evaluation of the Rat Model of Harmful Fluid Retention in the Upper Jiao", *World Journal of Integrated Traditional and Western Medicine*, vol. 10, no. 4, pp. 767-770, 2015.

[9] Y. Wang, M. Niu, G. L. Jia et al., "Untargeted Metabolomics Reveals Intervention Effects of Total Turmeric Extract in a Rat Model of Nonalcoholic Fatty Liver Disease", *Evidence-Based Complementary and Alternative Medicine*, vol. 2016, Article ID 8495953, 12pages, 2016.

[10] X. H. Xia, Y. Y. Yuan, and M. Liu, "The assessment of the chronic hepatotoxicity induced by *Polygoni Multiflori Radix* in rats: A pilot study by using untargeted metabolomics method", *Journal of Ethnopharmacology*, vol. 203, pp. 182-190, 2017.

[11] H. Bi, F. Li, K. W. Krausz et al., "Targeted Metabolomics of Serum Acylcarnitines Evaluates Hepatoprotective Effect of Wuzhi Tablet (*Schisandra sphenanthera* Extract) against Acute Acetaminophen Toxicity", *Evidence-Based Complementary and Alternative Medicine*, vol. 2013, Article ID 985257, 13 pages, 2013.

[12] J. López-Ibáñez, F. Pazos, and M. Chagoyen, "MBROLE 2.0-Functional enrichment of chemical compounds", *Nucleic Acids Research*, vol. 44, no. W1, pp. W201-W204, 2016.

[13] Y. Nan, X. H. Zhou, Q. Liu et al., "Serum metabolomics strategy for understanding pharmacological effects of Shen Qi pill acting on kidney yang deficiency syndrome", *Journal of Chromatography B-Analytical Technology in the Biomedical and Life Sciences*, vol. 1026, pp. 217-226, 2016.

[14] L. Zhao, D. Wu, M. Sang et al., "Stachydrine ameliorates isoproterenol-induced cardiac hypertrophy and fibrosis by suppressing inflammation and oxidative stress through inhibiting NF-κB

and JAK/STAT signaling pathways in rats", *International Immunopharmacology*, vol. 48, pp. 102-109, 2017.

[15] Y. H. Chen, H. B. Hu, Y. Li, Y. Q. Mao, and J. H. Zang, "Preparation of Pulmonary Fibrosis Rat Model by Modified Endotracheal Intubation with Medicinal Infusion", *World Journal of Integrated Traditional and Western Medicine*, vol. 9, no. 7, pp. 714-722, 2014.

[16] H. Kamiguchi, M. Yamaguchi, M. Murabayashi, I. Mori, and A. Horinouchi, "Method development and validation for simultaneous quantitation of endogenous hippuric acid and phenylacetylglycine in rat urine using liquid chromatography coupled with electrospray ionization tandem mass spectrometry", *Journal of Chromatography B-Analytical Technology in the Biomedical and Life Sciences*, vol. 1035, pp. 76-83, 2016.

[17] H. Kamiguchi, M. Murabayashi, I. Mori, A. Horinouchi, and K. Higaki, "Biomarker discovery for drug-induced phospholipidosis: phenylacetylglycine to hippuric acid ratio in urine and plasma as potentiall markers", *Biomarkers*, vol. 22, no. 2, pp. 178-188, 2017.

[18] W. Krause, M. Halminski, and L. McDonald, "Biochemical and neuropsychological effects of elevated plasma phenylalanine in patients with phenylketonuria", *Journal of Clinical Investigation*, vol. 75, no. 1, pp. 40-48, 1985.

[19] L. J. van Loon, W. H. Saris, H. Verhagen, and A. J. Wagenmakers, "Plasma insulin responses after ingestion of different amino acid or protein mixtures with carbohydrate", *American Journal of Clinical Nutrition*, vol. 72, no. 1, pp. 96-105, 2000.

[20] K. H. Schulpis, J. Tjamouranis, G. A. Karikas, H. Michelakakis, and S. Tsakiris, "In vivo effects of high phenylalanine blood levels on Na^+, K^+-ATPase, Mg^{2+}-ATPase activities and biogenic amine concentrations in phenylketonuria", *Clinical ~~Biochemist Reviews~~Biochemistry*, vol. 35, no. 4, pp. 281-285, 2002.

[21] E. V. Lopatina, A. V. Kipenko, N. A. Pasatetskaya, V. A. Penniyaynen, and B. V. Krylov, "Modulation of the transducer function of Na^+, K^+-ATPase: new mechanism of heart remodeling", *Canadian Journal of Physiology & Pharmacology*, vol. 94, no. 10, pp. 1110-1116, 2016.

[22] T. N. Rindler, I. Dostanic, V. M. Lasko et al., "Knockout of the Na, K-ATPase α_2-isoform in the cardiovascular system does not alter basal blood pressure but prevents ACTH-induced hypertension", *Ajp Heart & Circulatory Physiology*, vol. 301, no. 4, pp. H1396-1404, 2011.

[23] H. N. Lee, J. K. Kundu, Y. N. Cha, and Y. J. Surh, "Resolvin D1 stimulates efferocytosis

through p50/p50-mediated suppression of tumor necrosis factor-α expression”, *Journal of Cell Science*, vol. 126, no. 17, pp. 4037-4047, 2013.

[24] F. Xing, X. Hu, J. Jiang, Y. Ma, and A. Tang, “A meta-analysis of low-dose dopamine in heart failure”, *International Journal of Cardiology*, vol. 222, pp. 1003-1011, 2016.

[25] T. Ito, S. Schaffer, and J. Azuma, “The effect of taurine on chronic heart failure: actions of taurine against catecholamine and angiotensin II”, *Amino Acids*, vol. 46, no. 1, pp. 111-119, 2014.

[26] L. P. Ardisson, B. P. Rafacho, P. P. Santos et al., “Taurine attenuates cardiac remodeling after myocardial infarction”, *International Journal of Cardiology*, vol. 168, no. 5, pp. 4925-4926, 2013.

[27] Y. Li, J. M. Arnold, M. Pampillo, A. V. Babwah, and T. Peng, “Taurine prevents cardiomyocyte death by inhibiting NADPH oxidase-mediated calpain activation”, *Free Radical Biology & Medicine*, vol. 46, no. 1, pp. 51-61, 2009.

[28] T. J. Mac Cormack, N. I. Callaghan, A. V. Sykes, and W. R. Driedzic, “Taurine depresses cardiac contractility and enhances systemic heart glucose utilization in the cuttlefish, Sepia officinalis”, *Journal of Comparative Physiology B*, vol. 186, no. 2, pp. 215-227, 2016.

[29] X. Wu, Y. Yang, and D. Huang, “Effect of aqueous extract of *Lepidiunm apetalum* on dog’s left ventricular function”, *Journal of Chinese Medicinal Materials*, vol. 21, no. 5, pp. 243-245, 1998.

[30] K. P. Patel, F. J. Luo, N. S. Plummer, T. H. Hostetter, and T. W. Meyer, “The production of *p*-cresol sulfate and indoxyl sulfate in vegetarians versus omnivores”, *Clinical Journal of the American Society of Nephrology*, vol. 7, no. 6, pp. 982-988, 2012.

[31] E. Schepers, G. Glorieux, and R. Vanholder, “The gut: the forgotten organ in uremia?”, *Blood Purification*, vol. 29, no. 2, pp. 130-136, 2010.

[32] H. A. Mutsaers, P. Caetano-Pinto, A. E. Seegers et al., “Proximal tubular efflux transporters involved in renal excretion of *p*-cresyl sulfate and *p*-cresyl glucuronide: Implications for chronic kidney disease pathophysiology”, *Toxicology In Vitro*, vol. 29, no. 7, pp. 1868-1877, 2015.

[33] J. F. Winchester, T. H. Hostetter, and T. W. Meyer, “*p*-Cresol sulfate: further understanding of its cardiovascular disease potential in CKD”, *American Journal of Kidney Diseases*, vol. 54, no. 5, pp. 792-794, 2009.

[34] C. Y. Sun, H. H. Hsu, and M. S. Wu, “*p*-Cresol sulfate and indoxyl sulfate induce similar cellular inflammatory gene expressions in cultured proximal renal tubular cells”, *Nephrology Dialysis Transplantation*, vol. 28, no. 1, pp. 70-78, 2013.

[35] L. Wang, A. L. Cao, Y. F. Chi et al., "You-gui Pill ameliorates renal tubulointerstitial fibrosis via inhibition of TGF-β/Smad signaling pathway", *Journal of Ethnopharmacology*, vol. 169, no. 3, pp. 229-238, 2015.

[36] W. Zhang, W. Wang, H. Yu et al., "Interleukin 6 underlies angiotensin II-induced hypertension and chronic renal damage", *Hypertension*, vol. 59, no. 1, pp. 136-144, 2012.

[37] S. L. Barker, J. Pastor, D. Carranza et al., "The demonstration of αKlotho deficiency in human chronic kidney disease with a novel synthetic antibody", *Nephrology Dialysis Transplantation*, vol. 30, no. 2, pp. 223-233, 2015.

[38] H. Han, J. Zhu, Z. Zhu et al., "*p*-Cresyl sulfate aggravates cardiac dysfunction associated with chronic kidney disease by enhancing apoptosis of cardiomyocytes", *Journal of the American Heart Association: Cardiovascular and Cerebrovascular Disease*, vol. 4, no. 6, pp. 1-11, 2015.

[39] M. Yisireyili, H. Shimizu, S. Saito et al., "Indoxyl sulfate promotes cardiac fibrosis with enhanced oxidative stress in hypertensive rats", *Life Sciences*, vol. 92, pp. 1180-1185, 2013.

[40] K. Yang, X. Xu, L. Nie et al., "Indoxyl sulfate induces oxidative stress and hypertrophy in cardiomyocytes by inhibiting the AMPK/UCP2 signaling pathway", *Toxicology Letters*, vol. 234, no. 2, pp. 110-119, 2015.

Highlights

1. A novel fraction method based on compound polarity and type was employed to split the extract of traditional Chinese medicine.

2. The metabolomics approach and split fractions of extract were utilized in combination to illustrate pathologic changes of Chinese medicine syndrome and action mechanisms of traditional Chinese medicine.

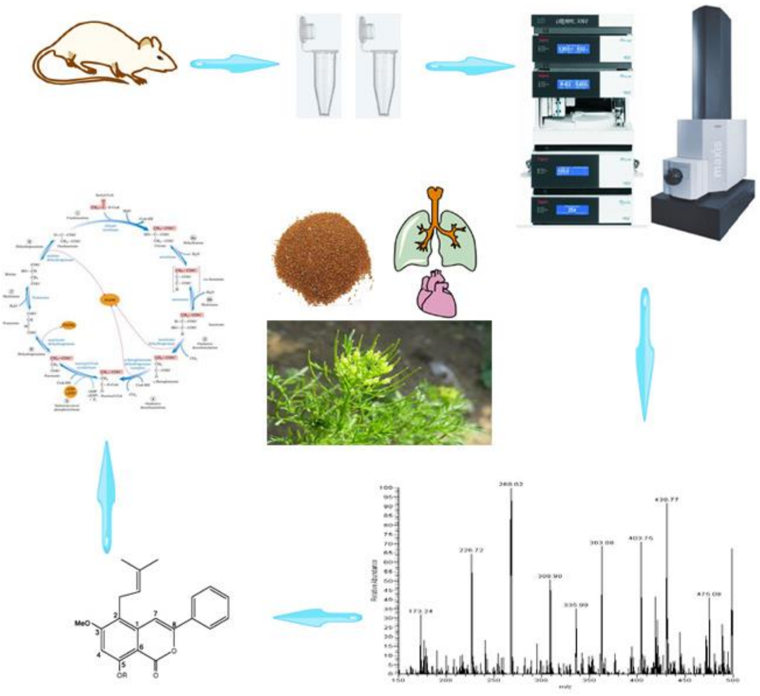

GRAPHICAL ABSTRACT

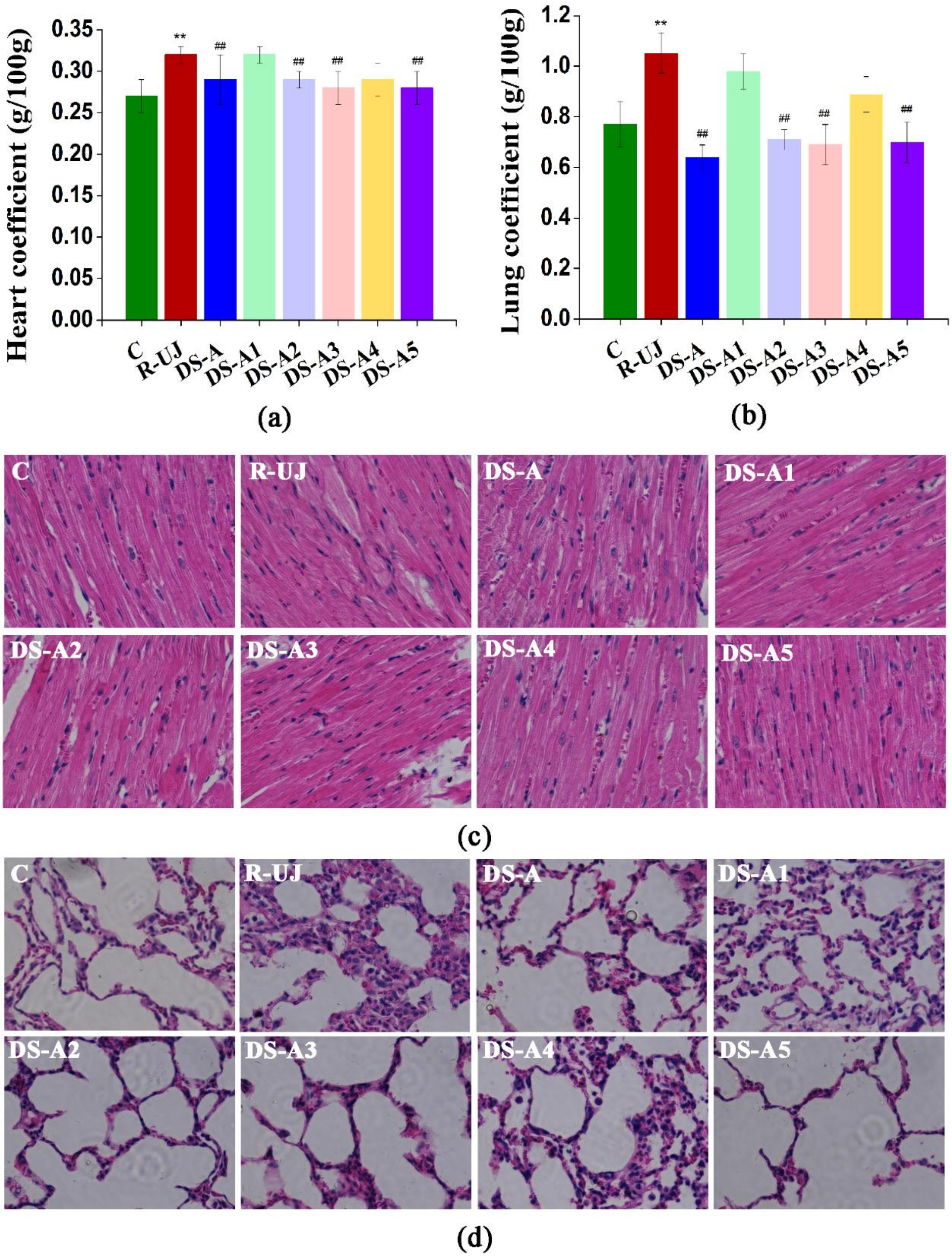

FIGURE 1: Organ coefficients and histopathological examination (Magnification 400×) in C, R-UJ model and DS-treated groups: heart coefficient (a), lung coefficient (b), HE stained slices of heart (c) and lung (d). *P < 0.05, **P < 0.01, compared with the control group; #P < 0.05, ##P < 0.01, compared with the model group.

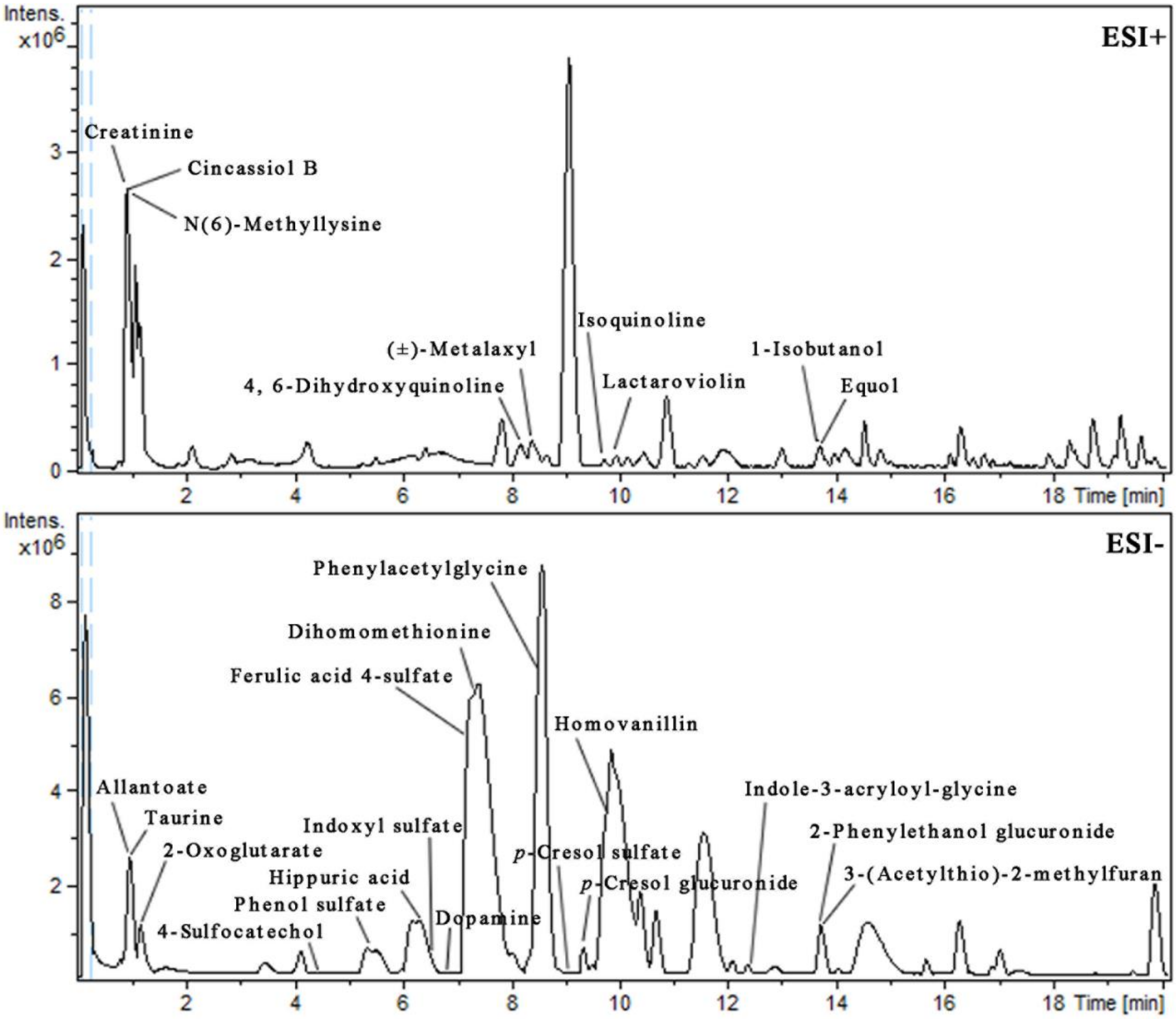

FIGURE 2: Representative base peak chromatograms obtained from urine in ESI$^+$ and ESI$^-$ mode.

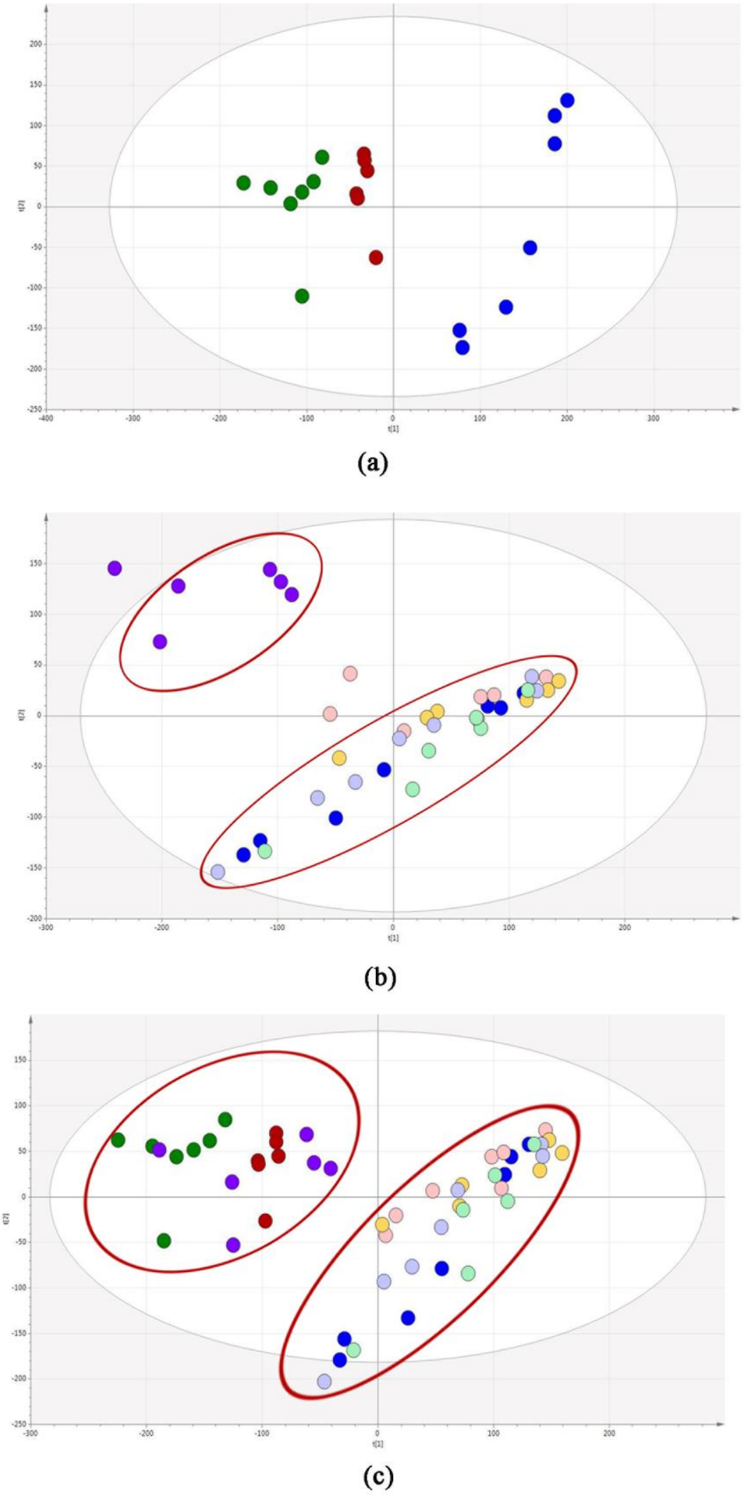

C group R-UJ group DS-A group DS-A1 group

DS-A2 group DS-A3 group DS-A4 group DS-A5 group

FIGURE 3: Multivariate data analysis: PCA score scatter plot obtained from C, R-UJ model and DS-A group (a); PCA score scatter plot obtained from all DS-treated groups (b); PCA score scatter plot obtained from all groups (c).

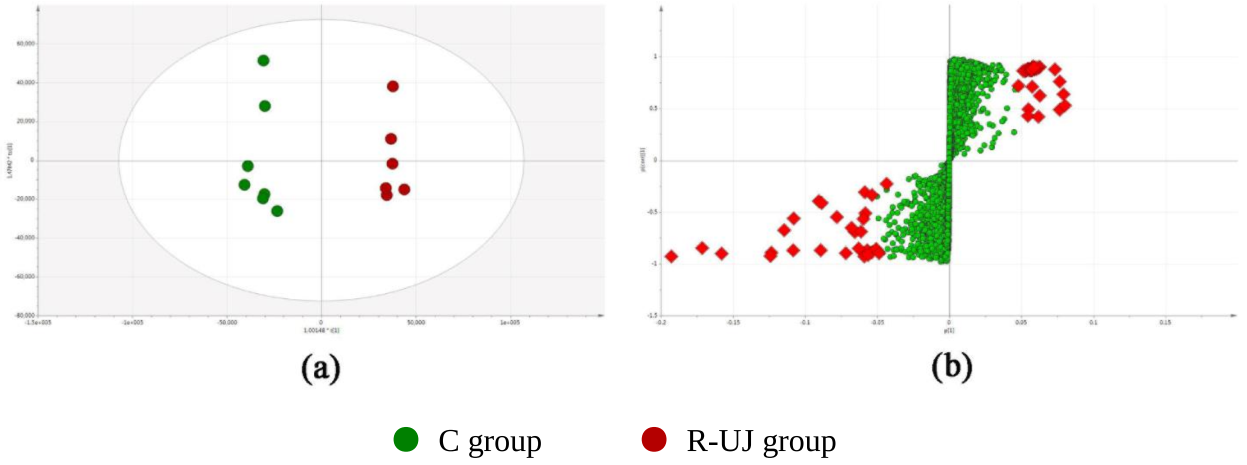

FIGURE 4: OPLS-DA score scatter plot obtained from C versus R-UJ model group (a), S-plot of OPLS-DA for R-UJ model group (b).

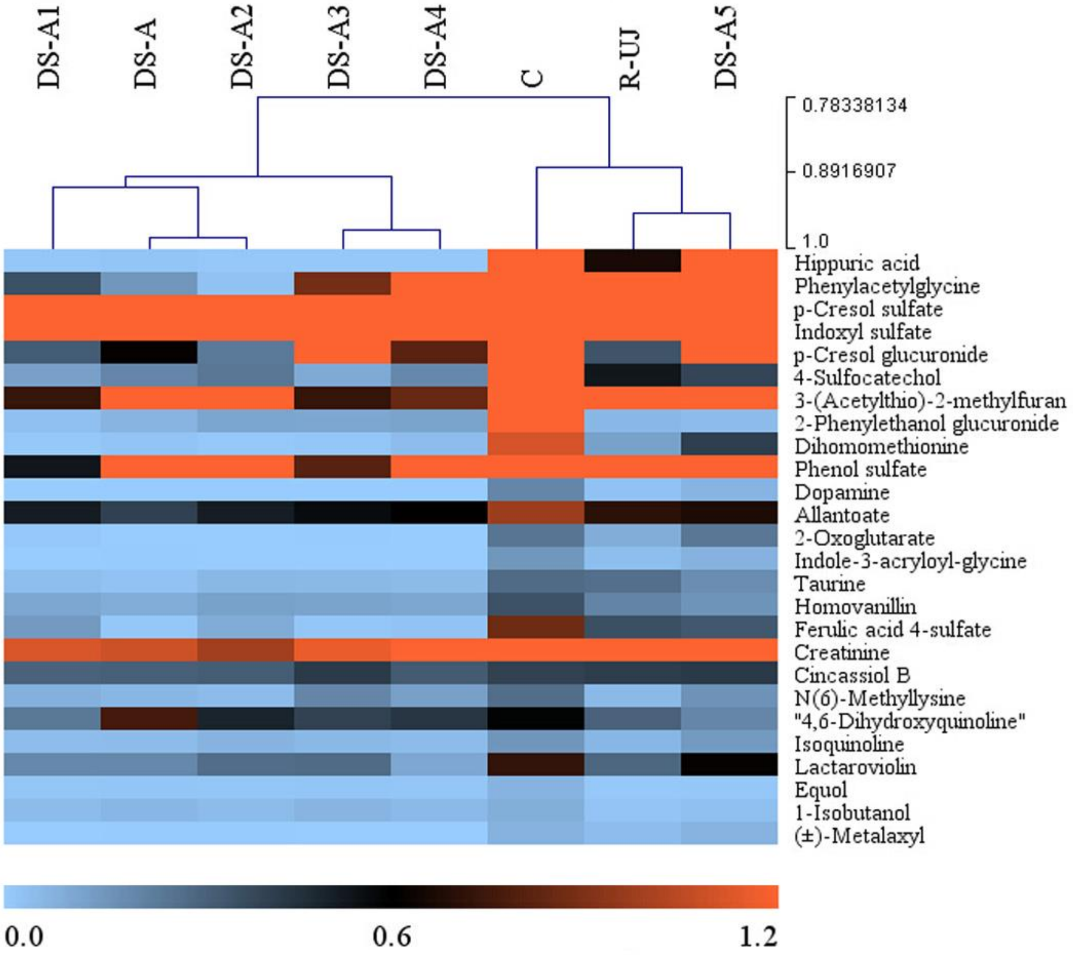

FIGURE 5: Heat map of the 26 potential biomarkers in C, R-UJ, DS-A, DS-A1, DS-A2, DS-A3, DS-A4 and DS-A5 group. The colors changing from blue to orange indicate more metabolites.

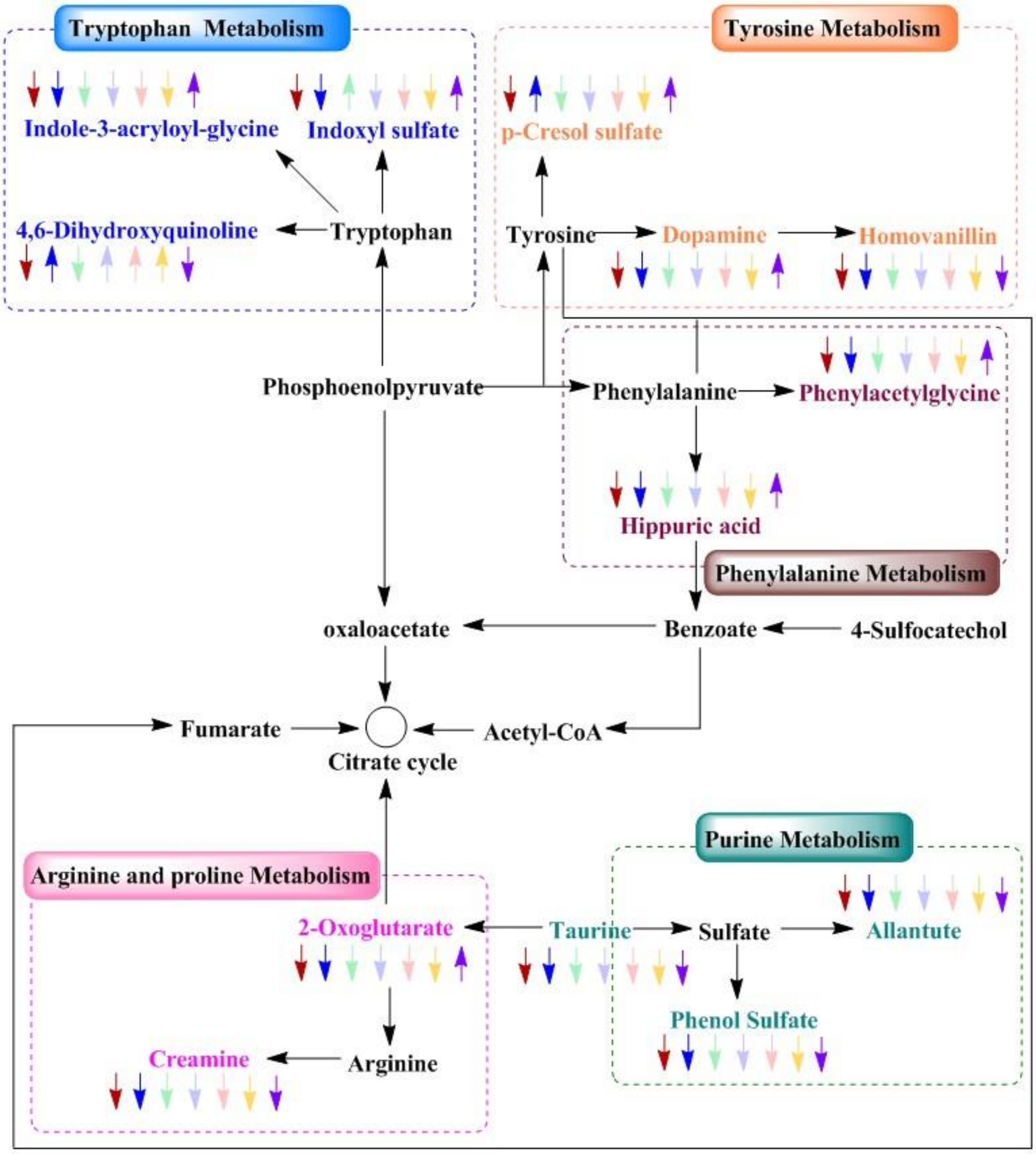

↓ / ↑ represents R-UJ model group compared with the control group;

↓ / ↑ , ↓ / ↑ , ↓ / ↑ , ↓ / ↑ , ↓ / ↑ , ↓ / ↑ represents DS-A, DS-A1, DS-A2, DS-A3, DS-A4 and DS-A5 group compared with R-UJ model group respectively.

FIGURE 6: Metabolic network of the significantly changed metabolites in urine of R-UJ rat.

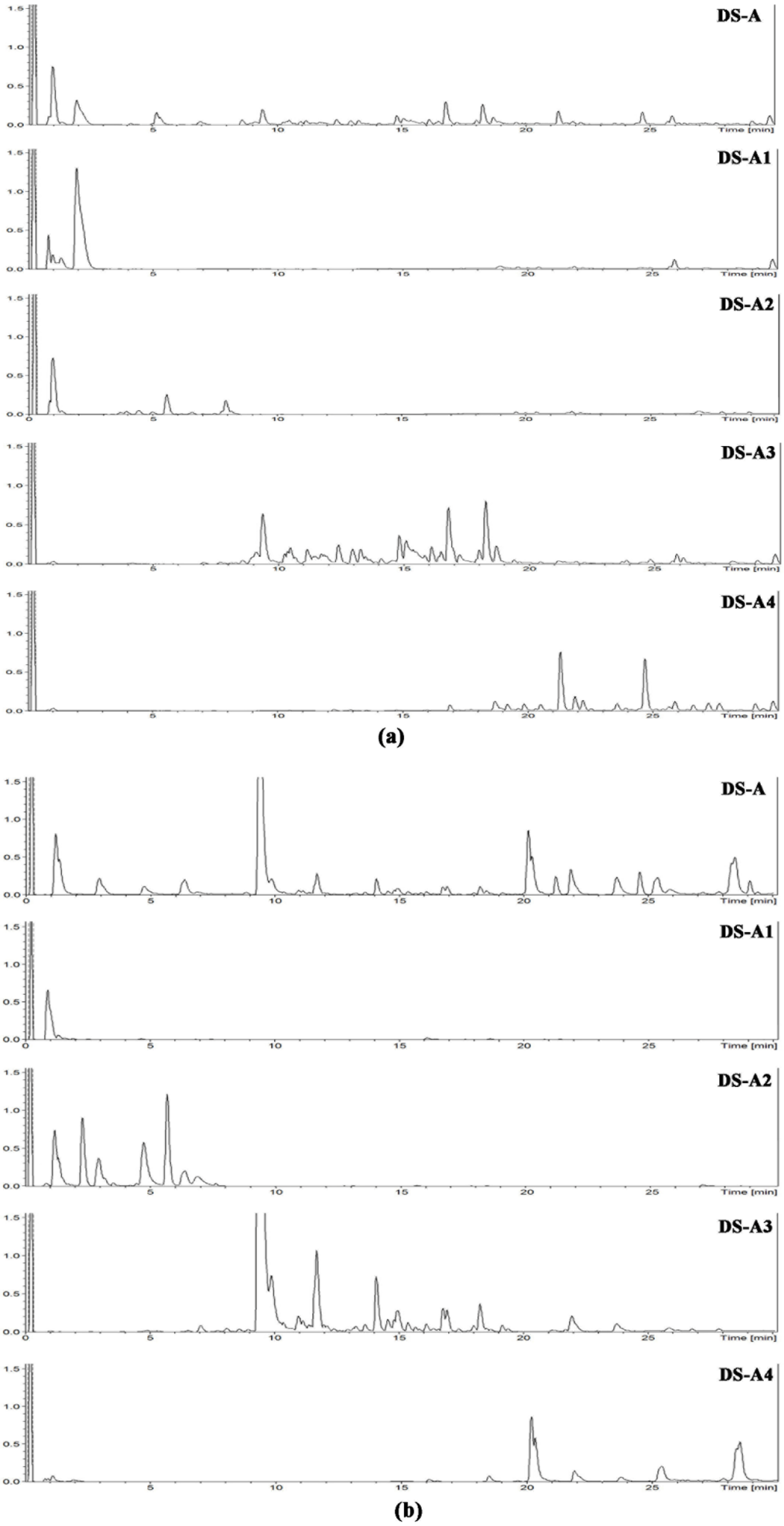

FIGURE S. 1: Base peak chromatograms of DS-A and its fractions in ESI^- (a) and ESI^+ (b) mode.

TABLE 1: Potential biomarkers related to R-UJ model.

Mode	No.	Name	Formula	Determined *m/z*	Ion form	t_R (min)	Trend
ESI$^-$	1	Hippuric acid	$C_9H_9NO_3$	178.0510	$[M-H]^-$	6.4	↓* ↓# ↓# ↓# ↓# ↓# ↑##
	2	Phenylacetylglycine	$C_{10}H_{11}NO_3$	192.0665	$[M-H]^-$	8.4	↓ ↓## ↓## ↓## ↓## ↓# ↑
	3	*p*-Cresol sulfate	$C_7H_8O_4S$	187.0070	$[M-H]^-$	9.0	↓** ↑ ↓ ↓ ↓ ↓ ↑
	4	Indoxyl sulfate	$C_8H_7NO_4S$	212.0021	$[M-H]^-$	6.6	↓** ↓ ↑ ↓ ↓ ↓ ↑
	5	*p*-Cresol glucuronide	$C_{13}H_{16}O_7$	283.0825	$[M-H]^-$	9.4	↓** ↑ ↓ ↓ ↑ ↑ ↑
	6	4-Sulfocatechol	$C_6H_6O_5S$	188.9858	$[M-H]^-$	4.4	↓** ↓## ↓## ↓# ↓## ↓## ↓
	7	3-(Acetylthio)-2-methylfuran	$C_7H_8O_2S$	201.0224	$[M+FA-H]^-$	13.7	↓** ↑ ↓ ↓ ↓ ↓ ↓
	8	2-Phenylethanol glucuronide	$C_{14}H_{18}O_7$	297.0979	$[M-H]^-$	13.7	↓** ↑ ↓ ↑ ↑ ↑ ↓
	9	Dihomomethionine	$C_7H_{15}NO_2S$	222.0802	$[M+FA-H]^-$	7.4	↓** ↓ ↓ ↓ ↓ ↓ ↑#
	10	Phenol sulfate	$C_6H_6O_4S$	172.9911	$[M-H]^-$	5.4	↓** ↓# ↓## ↓ ↓## ↓## ↓
	11	Dopamine	$C_8H_{11}NO_2$	134.0603	$[M-H_20-H]^-$	6.8	↓** ↓ ↓ ↓ ↓ ↓ ↑#
	12	Allantoate	$C_4H_8N_4O_4$	157.0361	$[M- H_20-H]^-$	1.0	↓* ↓# ↓ ↓ ↓ ↓ ↓
	13	2-Oxoglutarate	$C_5H_6O_5$	145.0134	$[M-H]^-$	1.2	↓** ↓# ↓ ↓# ↓# ↓# ↑#
	14	Indole-3-acryloyl-glycine	$C_{13}H_{14}N_2O_4$	243.0770	$[M-H_20-H]^-$	12.4	↓** ↓## ↓## ↓## ↓## ↓## ↑#
	15	Taurine	$C_2H_7NO_3S$	124.0065	$[M-H]^-$	1.0	↓ ↓## ↓## ↓## ↓## ↓## ↓#
	16	Homovanillin	$C_9H_{10}O_3$	165.0551	$[M-H]^-$	9.8	↓** ↓# ↓# ↓ ↓# ↓# ↓
	17	Ferulic acid 4-sulfate	$C_{10}H_{10}O_7S$	273.0069	$[M-H]^-$	7.2	↓** ↓# ↓ ↓ ↓# ↓# ↓
ESI$^+$	18	Creatinine	$C_4H_7N_3O$	114.0664	$[M+H]^+$	1.0	↓ ↓ ↓ ↓# ↓ ↓ ↓
	19	Cincassiol B	$C_{20}H_{32}O_8$	212.1033	$[M+Na+H]^{2+}$	1.0	↑ ↓ ↓ ↓ ↑ ↓ ↑
	20	N(6)-Methyllysine	$C_7H_{16}N_2O_2$	143.1181	$[M-H_20+H]^+$	1.0	↓** ↑ ↑ ↓ ↑ ↑ ↑
	21	4, 6-Dihydroxyquinoline	$C_9H_7NO_2$	162.0550	$[M+H]^+$	8.2	↓ ↑# ↓ ↑ ↑ ↑ ↓
	22	Isoquinoline	C_9H_7N	130.0651	$[M+H]^+$	9.7	↓* ↓ ↓ ↑ ↓ ↓ ↑
	23	Lactaroviolin	$C_{15}H_{14}O$	233.0923	$[M+Na]^+$	9.8	↓* ↓ ↓ ↓ ↓ ↓ ↑
	24	Equol	$C_{15}H_{14}O_3$	243.1018	$[M+H]^+$	13.6	↓* ↑ ↑ ↑ ↑ ↑ ↑
	25	1-Isobutanol	$C_{21}H_{27}NO_{10}$	436.1603	$[M-H_20+H]^+$	13.6	↓ ↑ ↑ ↑ ↑ ↑ ↑
	26	(±)-Metalaxyl	$C_{15}H_{21}NO_4$	280.1543	$[M+H]^+$	8.4	↓ ↓ ↓ ↓ ↓ ↓ ↑

*$P < 0.05$, **$P < 0.01$, compared with the control group; #$P < 0.05$, ##$P < 0.01$, compared with the model group.

↓ / ↑ represents R-UJ model group compared with the control group;

↓ / ↑ , ↓ / ↑ , ↓ / ↑ , ↓ / ↑ , ↓ / ↑ , ↓ / ↑ represents DS-A, DS-A1, DS-A2, DS-A3, DS-A4 and DS-A5 group compared with R-UJ model group respectively.

TABLE 2: Pathway enrichment analysis of perturbed metabolites in R-UJ rats based on MBRole database.

Label	*P*-value [a]	Related compounds
Phenylalanine metabolism	0.006	Phenylacetylglycine, Hippuric acid
Tyrosine metabolism	0.015	Dopamine, Homovanillin
Neuroactive ligand-receptor interaction	0.041	Dopamine, Taurine

[a] *P*-value is obtained from analysis of MBRole.
